# Recent Progress in Heterocycle Synthesis: Cyclization Reaction with Pyridinium and Quinolinium 1,4-Zwitterions

**DOI:** 10.3390/molecules28073059

**Published:** 2023-03-29

**Authors:** Zhen-Hua Wang, Yong You, Jian-Qiang Zhao, Yan-Ping Zhang, Jun-Qing Yin, Wei-Cheng Yuan

**Affiliations:** Innovation Research Center of Chiral Drugs, Institute for Advanced Study, Chengdu University, Chengdu 610106, China

**Keywords:** pyridinium, quinolinium, 1,4-zwitterions, cyclization reactions, heterocycles

## Abstract

Heteroarene 1, n-zwitterions are powerful and versatile building blocks in the construction of heterocycles and have received increasing attention in recent years. In particular, pyridinium and quinolinium 1,4-zwitterions have been widely studied and used in a variety of cyclization reactions due to their air stability, ease of use, and high efficiency. Sulfur- and nitrogen-based pyridinium and quinolinium 1,4-zwitterions, types of emerging heteroatom-containing synthons, have attracted much attention from chemists. These 1,4-zwitterions, which contain multiple reaction sites, have been successfully used in the synthesis of three- to eight-membered cyclic compounds over the last decade. In this review, we present the exciting progress made in the field of cyclization reactions of sulfur- and nitrogen-based pyridinium and quinolinium 1,4-zwitterions. Moreover, the mechanistic insights, the transition states, some synthetic applications, and the challenges and opportunities are also discussed. We hope to provide an overview for synthetic chemists who are interested in the heterocycle synthesis from cyclization reaction with pyridinium and quinolinium 1,4-zwitterions pyridinium and quinolinium 1,4-zwitterions.

## 1. Introduction

Heterocycles are ubiquitous cores in natural products, bioactive molecules, functional materials, and therapeutic leads [1,2,3,4]. Chemists are becoming increasingly focused on developing efficient synthetic approaches for heterocyclic frameworks, where minimizing the number of synthetic steps, maximizing synthesis efficiency, and reducing side reactions are important evaluation criteria [5,6,7,8,9,10,11,12,13,14,15]. Among various synthetic strategies, dipolar cyclization reactions have become one of the most favored methods for the construction of functionalized heterocyclic compounds [16,17,18,19,20]. The development of new types of dipoles and the exploration of their potential applications in cyclization reactions are new challenges in the field of modern organic chemistry [21,22,23,24,25,26,27].

**Figure 1:**
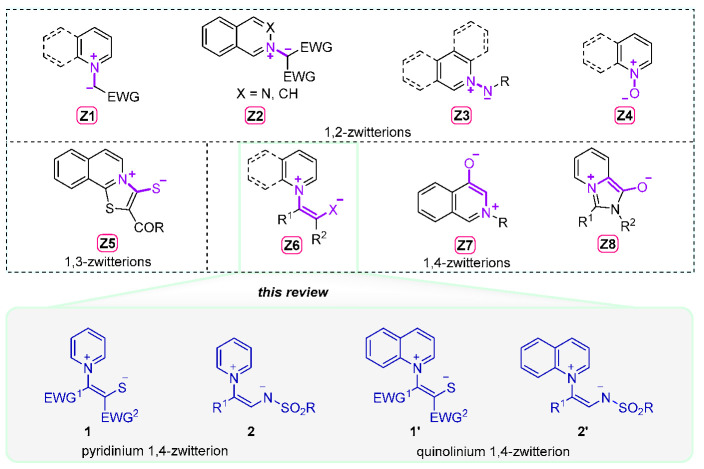
Overview of the pyridinium and (iso)quinolinium 1,n-zwitterions.

**Figure 2:**
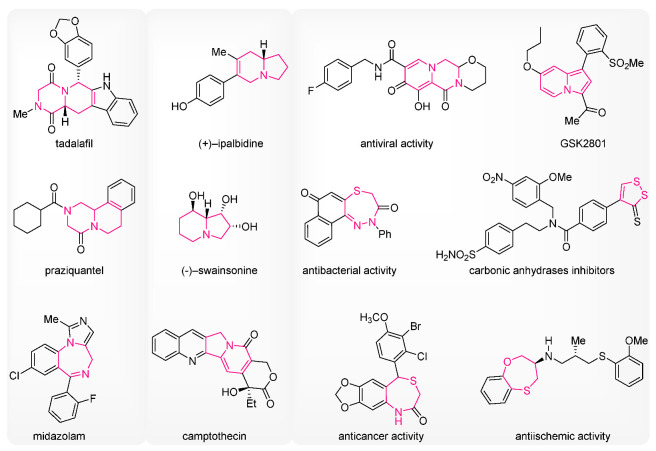
Representative marketed drugs, natural products, and bioactive molecules.

The application of heteroarene 1,n-zwitterions as powerful and versatile building blocks allows rapid synthesis of polyheterocyclic scaffolds that can be found in natural products, biologically active synthetic substances, and clinical drugs [28,29,30]. In particular, pyridinium and (iso)quinolinium 1,n-zwitterions are an important class of highly active species for constructing functionalized heterocycles. Great progress has been made in recent decades regarding the development of pyridinium and (iso)quinolinium 1,n-zwitterions, which are frequently classified into 1,2-, 1,3-, and 1,4-zwitterions based on the distance between the cation and anion (Figure 1). 1,2-Zwitterions (**Z1**–**Z4**) typically act as formal 1,3-dipoles in cyclization processes to generate a diverse range of polyheterocyclic structures [31,32,33]. Isoquinolinium thiolates **Z5**, as representative 1,3-zwitterions, are effective cycloaddition partners and provide access to sulfur-bridged cyclic polycycles [34]. 1,4-Zwitterion **Z6,** summarized in this review, is known for its versatility in the synthesis of heterocyclic skeletons such as thiophene, dithiole, thiazine, thiadiazepine, thiazepine, oxathiepine, indolizine, pyrido[1,2-*a*]pyrazine, and (di)azepine, which are widely distributed in various natural products, designed molecules, and drugs. Numerous natural or designed molecules usually have pronounced bioactivities (Figure 2) [35,36,37,38,39,40,41,42,43,44,45]. The remaining 1,4-zwitterions (**Z7** and **Z8**) have also been used as formal 1,3-dipoles to construct nitrogen-containing frameworks, such as indolizines and bridged azacycles [46,47].

Pyridinium and quinolinium 1,4-zwitterions (**Z6**) are highly valued building blocks in the construction of heterocycles due to their air stability, ease of use, and efficiency. They are divided into two categories based on the type of negative ion: sulfur-based 1,4-zwitterions and nitrogen-based 1,4-zwitterions.

**Scheme 1:**
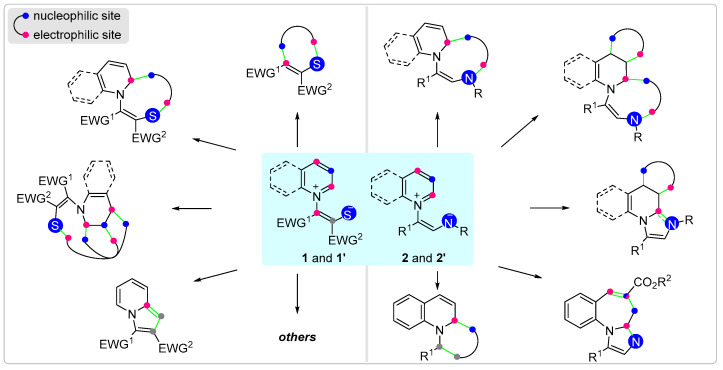
Common cyclization reaction with pyridinium and quinolinium 1,4-zwitterions, discussed in this review.

The synthesis of sulfur-based 1,4-zwitterions was first reported by Bazgir et al. in 2011 [48]. Despite this early report, their application in the construction of heterocycles has only been studied in recent years. At present, sulfur-based pyridinium and quinolinium 1,4-zwitterions have been successfully used in a range of formal cyclization reactions, including (2 + 3), (3 + n), (4 + n), (5 + n), and multistep cascade cyclization reactions (Scheme 1, left).

The first report of nitrogen-based 1,4-zwitterions was by Yoo et al. in 2014, who reported an Rh(II)-catalyzed reaction of 1-sulfonyl-1,2,3-triazole and pyridine to obtain isolable nitrogen-based 1,4-zwitterions [49]. Since then, the transformations involving dearomative cyclization have flourished (Scheme 1, right).

The aim of this review is to provide a comprehensive overview of the recent advancements in the transformation of pyridinium and quinolinium 1,4-zwitterions in the synthesis of heterocycles. At present, partial reactions of pyridinium and quinolinium 1,4-zwitterions have been selected as particular aspects, appearing in several published reviews and perspectives [20,31]. However, because of the explosive development of multifarious cyclization reactions involving pyridinium and quinolinium 1,4-zwitterions, these summaries cannot cover the latest achievements. In this context, a comprehensive and up-to-date overview of the application of pyridinium and quinolinium 1,4-zwitterions in the synthesis of heterocycles is highly desired.

The review is organized based on the categories of negative ions in pyridinium and quinolinium 1,4-zwitterions, which can be divided into sulfur-based and nitrogen-based types (Scheme 1). The annulation process is further classified based on the number of atoms of the final ring present in each fragment, designating the union of an m-atom fragment and an n-atom fragment as an (m + n) cyclization reaction. The purpose of this formalism is to make the skeletal analysis more convenient and it does not imply any mechanistic details.

## 2. Sulfur-Based Pyridinium and Quinolinium 1,4-Zwitterions

**Scheme 2:**
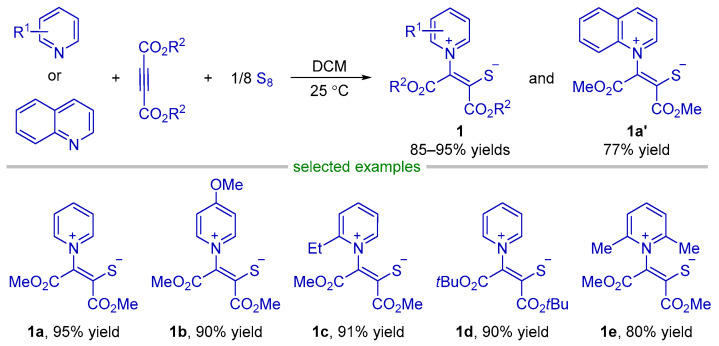
Synthesis of sulfur-based pyridinium and quinolinium 1,4-zwitterions.

The sulfur-based pyridinium and quinolinium 1,4-zwitterions reviewed in this review were discovered as early as 2011 by Bazgir et al. (Scheme 2) [48], though the applications of these molecules in the synthesis of heterocyclic scaffolds were not studied until 2019. The cyclization processes depicted in this paper are subdivided into formal (2 + 3), (3 + n), (4 + n), (5 + n), and multistep cascade cyclization reactions.

### 2.1. Formal (2 + 3) Cyclization

**Scheme 3:**
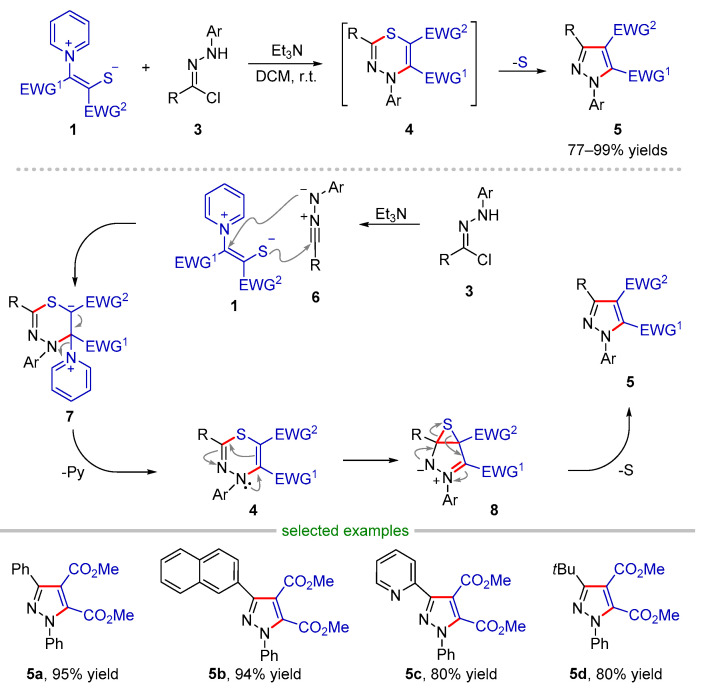
Formal (2 + 3) cyclization between sulfur-based pyridinium 1,4-zwitterions and hydrazonoyl chlorides.

In 2020, Zhai et al. conducted the formal (2 + 3) cyclization reaction between pyridinium 1,4-zwitterions **1** and hydrazonoyl chlorides **3** for the facile synthesis of fully substituted pyrazoles **5** (Scheme 3) [50]. According to the proposed reaction mechanism, the reaction proceeded via an unusual ((3 + 3) − 1) pathway. Hydrazonoyl chloride **3** reacted in situ with a base to generate the reactive nitrilimine **6**, which immediately reacted with pyridinium 1,4-zwitterion **1** following sequential S-nucleophilic addition, *N*-Michael addition, and retro-Michael addition/pyridine extrusion via reaction pathways, furnishing the key intermediate, 4*H*-1,3,4-thiadiazine **4**. The subsequent intramolecular nucleophilic addition of enamine to imine yielded intermediate **8**. Intermediate **8** could be converted into fully substituted pyrazole **5** via a desulfuration reaction. The developed method features a broad substrate scope, mild reaction conditions, and high yields.

### 2.2. Formal (3 + n) Cyclization

#### 2.2.1. Formal (3 + 2) Cyclization

**Scheme 4:**
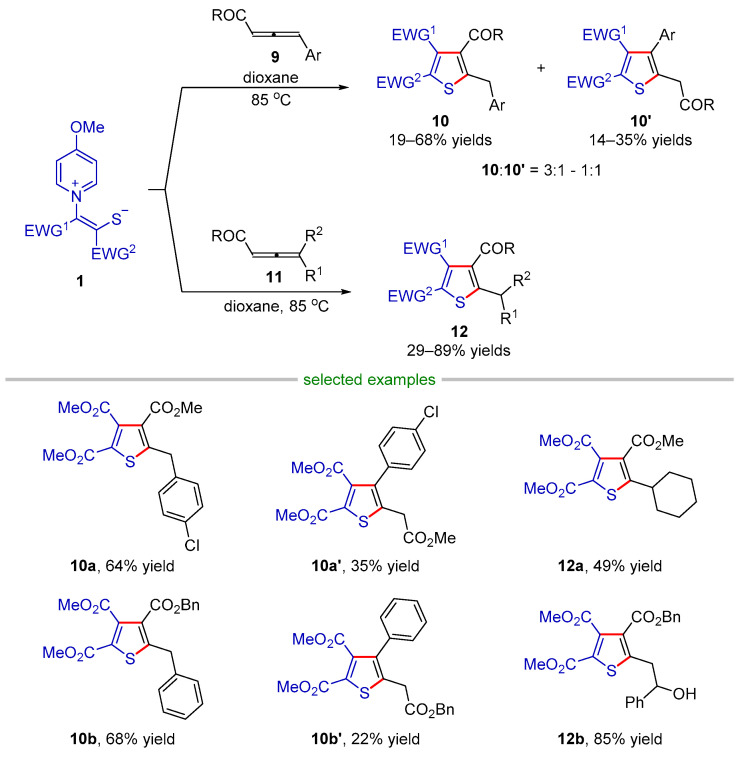
Formal (3 + 2) cyclization reaction of sulfur-based pyridinium 1,4-zwitterions with activated allenes.

**Scheme 5:**
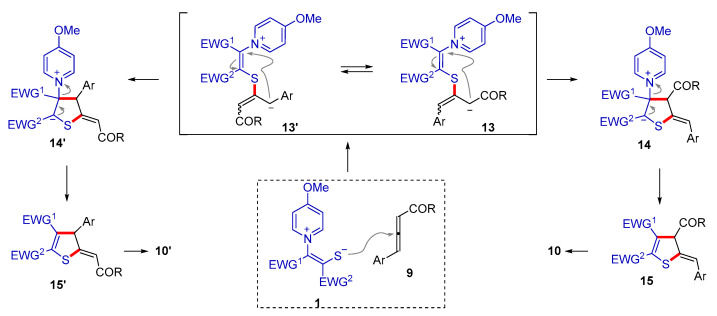
Proposed mechanism for (3 + 2) cyclization reaction between sulfur-based pyridinium 1,4-zwitterions and activated allenes.

In early 2020, annulations of pyridinium 1,4-zwitterions, and activated allenes were reported by Zhai, Wang, and Cheng et al. [51], who used pyridinium 1,4-zwitterions as three-carbon synthons to construct five-membered heterocyclic compounds. As illustrated in Scheme 4, the type of substituent presented a remarkable effect on the regioselectivity. When the reaction was conducted with *γ*-aryl-substituted allenoates **9**, a low level of regioselectivity was observed and major isomer **10** could be obtained in 19–68% yields. In contrast, when *γ*-alkyl-substituted allenoates **11** were used as the substrates, a highly regioselective cycloaddition reaction proceeded to yield the fully substituted thiophenes **12** in yields of up to 89%. Using this mechanism, it has been proposed that the *S*-Michael addition of pyridinium **1** to allenoates **9** results in the formation of intermediates **13** and **13′** (Scheme 5). This is followed by the intramolecular *C*-Michael addition of the carbanion located at the α-position of ester or benzyl position, yielding **14** and **14′**. The retro-Michael reaction results in the release of 4-MeO-pyridine, and this reaction is followed by a double bond isomerization reaction that yields two isomers (**10** and **10′**).

**Scheme 6:**
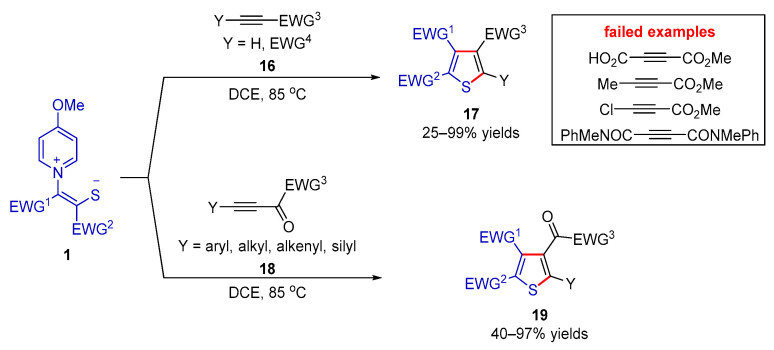
Formal (3 + 2) cyclization reaction between sulfur-based pyridinium 1,4-zwitterions and activated alkynes.

In the same year, Zhai et al. used sulfur-based pyridinium 1,4-zwitterion as a versatile building block to synthesize polysubstituted thiophenes [52]. The reactions between pyridinium 1,4-zwitterions **1** and activated alkynes **16** were accomplished in 1, 2-dichloroethane (DCE) at 85 °C via a (3 + 2) process, affording *tri*- and *tetra*-substituted thiophenes **17** in 25–99% yields (Scheme 6, top). The limitations in the substrate scope were explored, and it was observed that some modified alkynes were not compatible with the developed protocol. In the following year, an extension of this strategy was reported by Zhai et al. (Scheme 6, bottom) [53]. Various modified and activated alkynes **18** bearing aryl, alkenyl, alkyl, or silyl groups were used to conduct (3 + 2) annulation reactions with pyridinium 1,4-zwitterions **1**. The reaction proceeded smoothly to afford tetrasubstituted thiophenes **19** in 40–97% yields under the same reaction conditions. The developed approach has the features of being metal-free and catalyst-free.

**Scheme 7:**
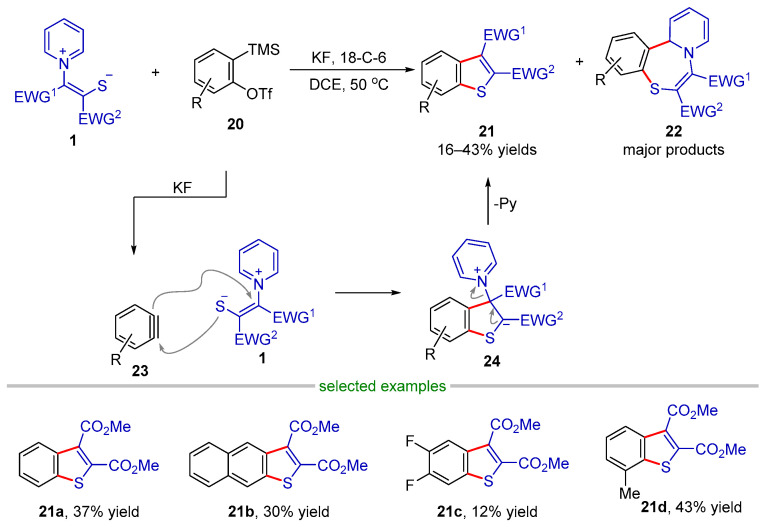
(3 + 2) Cyclization reaction between sulfur-based pyridinium 1,4-zwitterions and arynes formed in situ.

In 2020, Zhai’s group used *o*-(trimethylsilyl)phenyl triflate **20** and pyridinium 1,4-zwitterions **1** as substrates to conduct cyclization reactions. They reported that the reactions could follow two pathways (Scheme 7) [54]. The formal (5 + 2) cyclization reaction produced benzopyridothiazepines **22** as its major products. Although the (3 + 2) cyclization reaction was considered a side reaction, the results revealed that pyridinium 1,4-zwitterions could be used as powerful potential synthons to construct benzothiophenes **21**. In the developed protocol, benzothiophenes **21** could be obtained in up to 43% isolated yield.

**Scheme 8:**
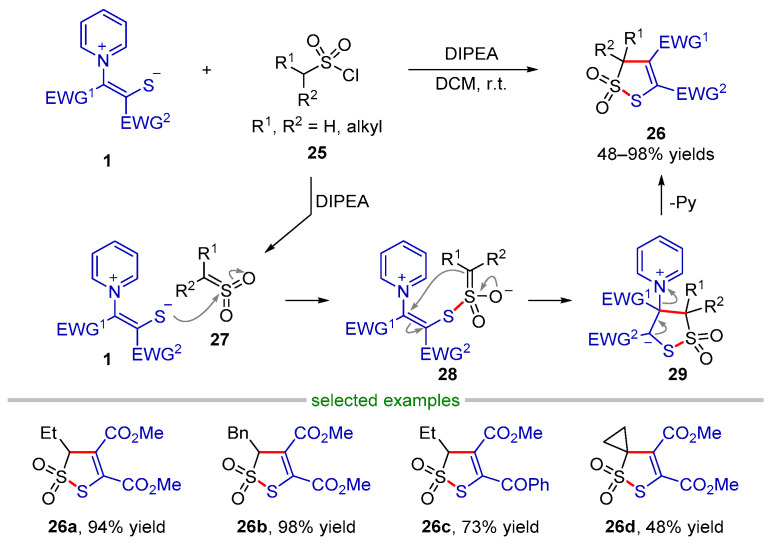
The (3 + 2) cyclization reaction of sulfur-based pyridinium 1,4-zwitterions and alkanesulfonyl chlorides.

In comparison to the numerous studies on the annulation of C=C and C≡C bonds, there are a limited number of examples of the (3 + 2) cyclization reaction between sulfur-based pyridinium 1,4-zwitterions and C=X or C≡X bonds (X = S, N). In 2020, Zhai et al. described the synthesis of 3*H*-1,2-dithiole 2,2-dioxides **26** through the (3 + 2) cyclization of pyridinium 1,4-zwitterions **1** with alkanesulfonyl chlorides **25** (as depicted in Scheme 8) [55]. The use of alkanesulfonyl chlorides **25** as precursors of sulfenes **27** allowed for the smooth transformation of the reaction in the presence of *N*,*N*-diisopropylethylamine (DIPEA), resulting in 3*H*-1,2-dithiole 2,2-dioxides **26** with yields ranging from 48% to 98%.

The reaction mechanism involves the transformation of alkanesulfonyl chloride **25** into sulfene **27** through the promotion of a selected base, which was attacked by sulfur anion of pyridinium 1,4-zwitterion to form sulfur–sulfur bonds. This is followed by a domino Michael/retro-Michael reaction that releases the pyridine group and yields the product **26**. In this paper, the authors also found that arylmethanesulfonyl chloride could react with pyridinium 1,4-zwitterions through a stepwise ((5 + 2) − 1) pathway.

**Scheme 9:**
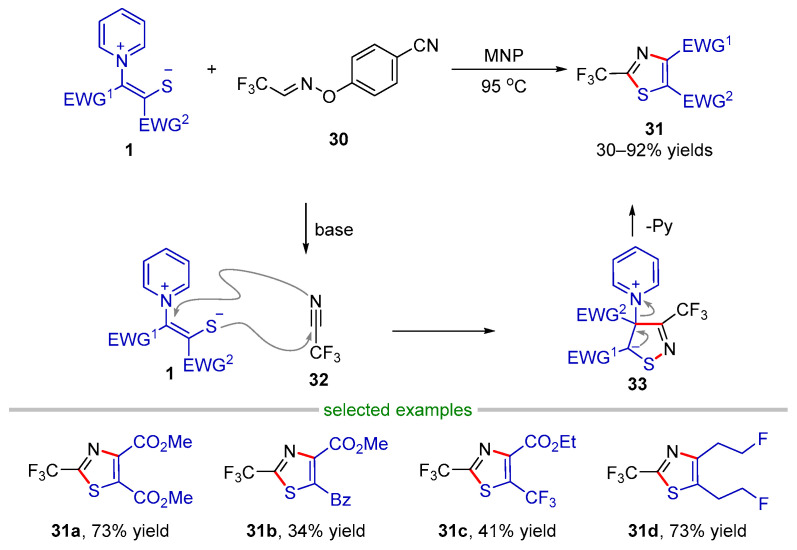
(3 + 2) Cyclization reaction between sulfur-based pyridinium 1,4-zwitterions and trifluoroacetaldehyde oxime.

More recently, Wen et al. investigated the (3 + 2) cycloaddition of pyridinium 1,4-zwitterion with trifluoroacetaldehyde *O*-(aryl)oxime (Scheme 9) [56]. The reaction, performed in *N*-methylpyrrolidone (NMP) at 95 °C, afforded the 2-trifluoromethyl 4,5-disubstituted thiazoles **31** in good-to-perfect yields (30–92% yield). The reaction mechanism was proposed. It was hypothesized that the treatment of oxime **30** with pyridine yielded CF_3_CN **32**. This reaction was followed by sequential *S*-nucleophilic addition and *N*-Michael reaction cascade that resulted in the formation of intermediate **33**. Finally, the retro-Michael reaction led to pyridine extrusion, and simultaneously furnished the desired products.

#### 2.2.2. Formal (3 + 3) Cyclization

**Scheme 10:**
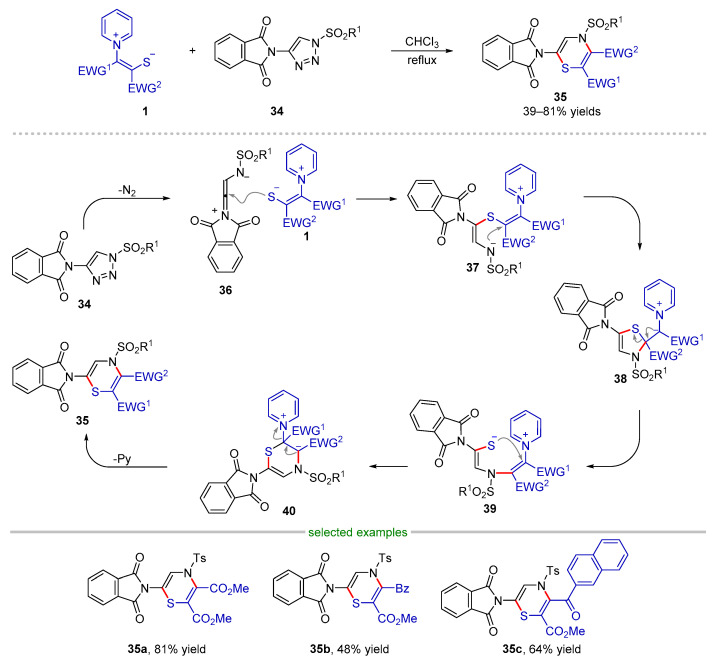
Formal (3 + 3) cyclization reaction of sulfur-based pyridinium 1,4-zwitterions with 4-NPhth substituted triazoles.

Formal (3 + 3) cyclization reactions belong to a class of important and powerful reactions that help synthesize six-membered heterocyclic rings. Li et al. devised a catalyst-free (3 + 3) cyclization strategy using pyridinium 1,4-zwitterions **1** to synthesize 1,4-thiazine derivatives **35** (Scheme 10) [57]. The reactions with 4-NPhth substituted triazoles **34** were carried out in CHCl_3_ under conditions of reflux, and moderate-to-good yields were observed. A possible mechanism was postulated to explain the selective formation of the 1,4-thiazine skeleton (Scheme 10, middle). Under optimal reaction conditions, 4-NPhth substituted triazoles **34** transformed into intermediate **36**, followed by the sequential *S*-nucleophilic addition and *N*-Michael reactions to yield thiazole intermediate **38**. Ring expansion resulted in the formation of intermediate **40** following retro-*S*-Michael reaction/*S*-Michael reaction. Finally, the corresponding product **35** was delivered through the elimination of the pyridine group.

**Scheme 11:**
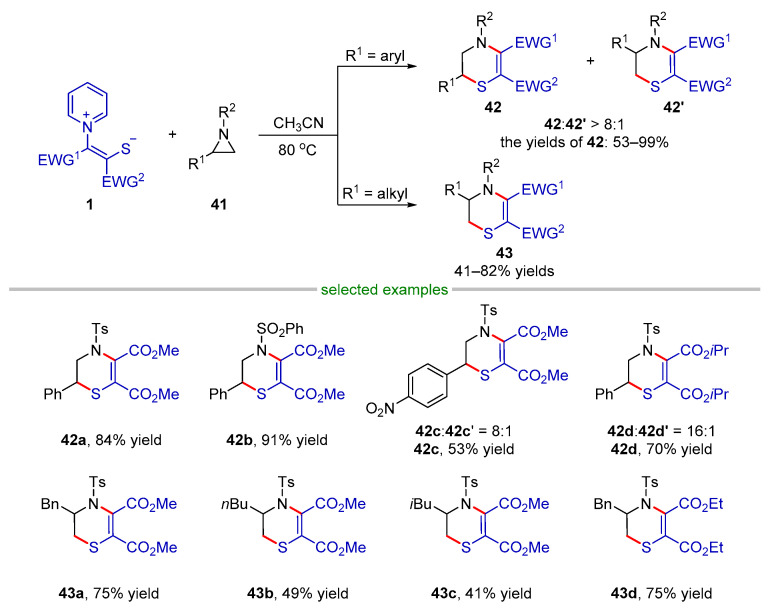
Formal (3 + 3) cyclization reaction of sulfur-based pyridinium 1,4-zwitterions and aziridines.

**Scheme 12:**
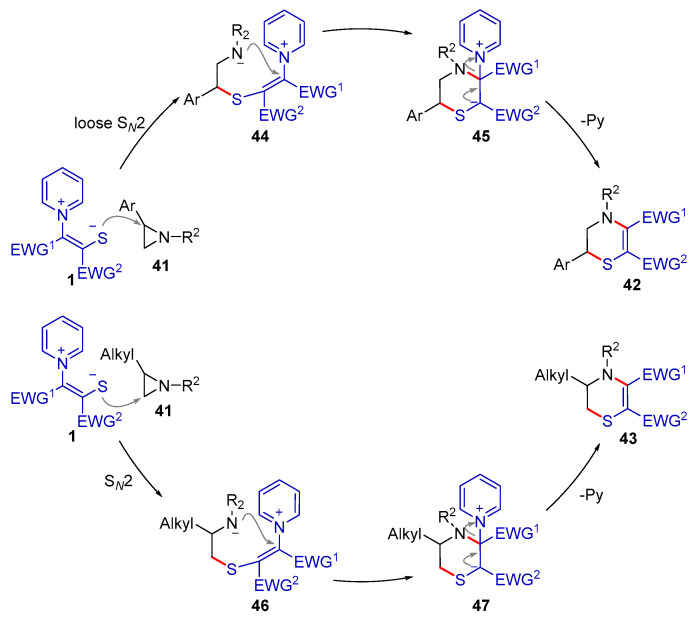
Reaction pathways for formal (3 + 3) cyclization reaction involving sulfur-based pyridinium 1,4-zwitterions, and aziridines.

Another example of the formal (3 + 3) cyclization of pyridinium 1,4-zwitterions **1** was reported by Chen et al. in 2022 (Scheme 11) [58]. The reaction with both alkyl- and aryl-substituted aziridines provided a wide range of functionalized 3,4-dihydro-2*H*-1,4-thiazines (**42** and **43**) in good-to-high yields with excellent levels of regioselectivity. Substrate scope was studied, and it was observed that the type of substituents on aziridines significantly affected the regioselectivity of the reaction. The authors proposed a mechanism to illustrate the origin of regioselectivity (Scheme 12). For 2-arylaziridine, the *S*-nucleophilic addition to the more sterically hindered site of the aziridine ring via a loose S*_N_*2 ring-opening process [59,60] lead to the formation of intermediate **44**. This reaction was followed by an *N*-Michael/retro-Michael reaction that yielded 1,4-thiazine **42**. In contrast, the ring-opening reaction of 2-alkylaziridine occurred at the less sterically hindered site via an S*_N_*2 pathway to yield intermediate **46**. This resulted in the formation of the corresponding product **43**. The protocol’s features include being catalyst- and base-free and having high regioselectivity.

#### 2.2.3. Formal (3 + 4) Cyclization

Seven-membered rings as integral subunits are ubiquitous in a wide variety of clinical drugs and bioactive natural products [61,62,63], and selectively synthesizing structurally diverse seven-membered rings remains an important pursuit in the field of organic synthetic chemistry [64,65,66,67]. The (3 + 4) cycloaddition reaction has attracted more and more attention for its diversity and high efficiency [68,69,70,71]. Formal (3 + 4) cyclization involving sulfur-based pyridinium 1,4-zwitterions has been used in the synthesis of seven-membered heterocyclic skeletons such as thiadiazepine, thiazepine, and oxathiepine.

**Scheme 13:**
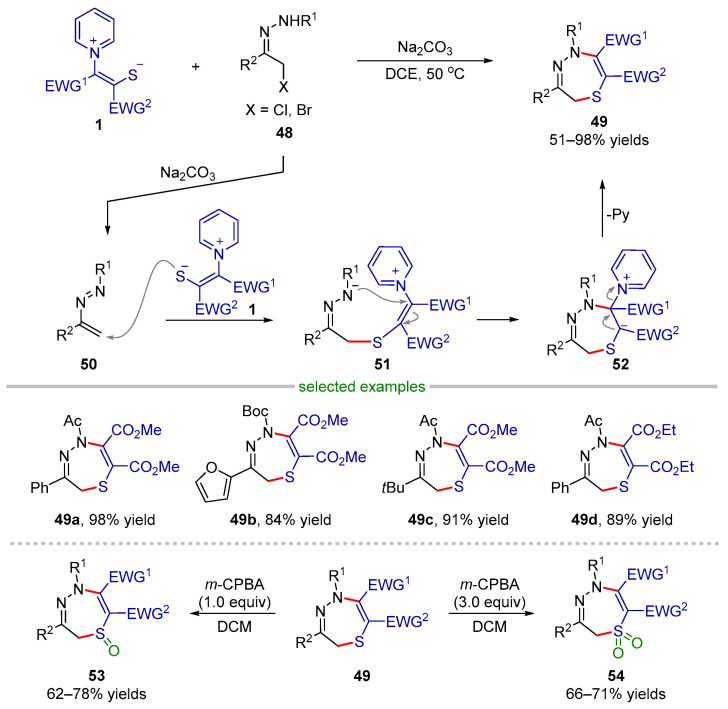
(3 + 4) Cyclization reaction involving sulfur-based pyridinium 1,4-zwitterions and α-halo hydrazones.

Zhai and Cheng et al. were the first to report a clever strategy for (3 + 4) cycloaddition reactions involving pyridinium 1,4-zwitterions (Scheme 13). Pyridinium 1,4-zwitterions **1** were selected as three-atom synthons to react with *α*-halo hydrazones **48**, leading to the formation of 1,4,5-thiadiazepine derivatives **49** in generally good-to-excellent yields (51–98%) [72]. For the reaction mechanism, azoalkenes **50** were generated in situ from *α*-halo hydrazones **48** in the presence of a base. The *S*-Michael addition, *N*-Michael addition, and retro-Michael addition reactions proceeded sequentially, resulting in the formation of 2,5-dihydro-1,4,5-thiadiazepines **49**. It is of note that the selective oxidation of **49** was also successfully established, in which sulfone **53** and sulfoxide **54** analogs could be produced in good, isolated yields (Scheme 13, bottom).

**Scheme 14:**
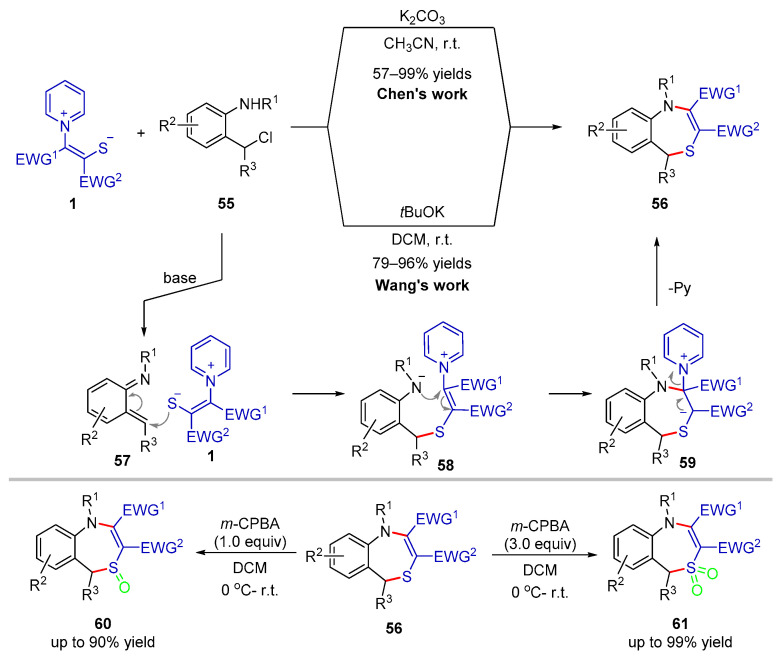
(3 + 4) Cyclization reaction between sulfur-based pyridinium 1,4-zwitterions and *N*-(*o*-chloromethyl)aryl amides.

In 2021, both Chen et al. and Wang et al. independently implemented (3 + 4) cycloaddition reactions between aza-*o*-quinone methides **57** (in situ generated from **55**) and pyridinium 1,4-zwitterions **1** (Scheme 14, top) [73,74]. Chen et al. selected K_2_CO_3_ as the optimal base to promote the reaction, and the reaction yielded functionalized benzo[*e*][1,4]thiazepines **56** in 57–99% yields [73]. In contrast, Wang et al. carried out the reaction between *N*-(*o*-chloromethyl)aryl amides **55** and pyridinium 1,4-zwitterions **1** in the presence of *t*BuOK in CH_2_Cl_2_ to obtain the corresponding products **56** in yields of up to 96% [74]. They proposed a similar reaction mechanism for the [4 + 3] annulation reaction. Treatment of *N*-(*o*-chloromethyl)aryl amide **55** with optimal base furnished aza-*o*-quinone methide **57,** which reacted with pyridinium 1,4-zwitterions **1** to yield intermediate **58**. The intramolecular *S*-Michael addition of **58** produced intermediate **59.** Finally, retro-Michael addition led to the formation of the desired product **56**. The selective oxidation of products was achieved by both research groups (Scheme 14, bottom).

**Scheme 15:**
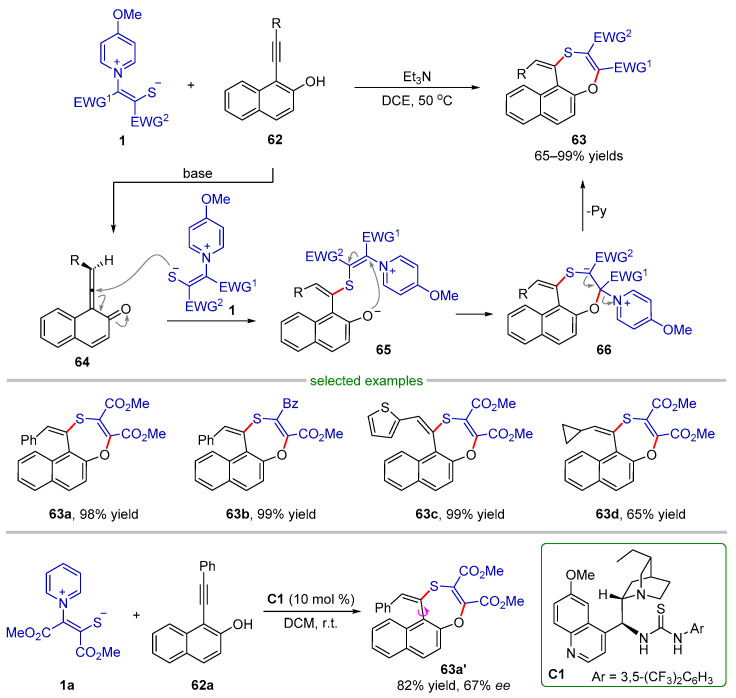
**A** (3 + 4) cyclization reaction between pyridinium 1,4-zwitterions and α-alkynylnaphthalen-2-ols.

A clever strategy for the synthesis of benzooxathiepines was devised by Cheng et al., who used Et_3_N as the base to enable (3 + 4) cycloaddition using pyridinium 1,4-zwitterions **1** and *ortho*-alkynyl aromatic phenols **62**. The reaction yielded aryl-fused 1,4-oxathiepines **63** in 65–99% yields (Scheme 15) [75]. In the proposed mechanism, *ortho*-alkynyl aromatic phenol **62** converted into vinylidene *ortho*-quinone methide **64** in the presence of an optimal base, and the intermediate **64** reacted with pyridinium 1,4-zwitterion **1** to produce intermediate **65** through *S*-nucleophilic addition. The intramolecular *O*-Michael addition of **65** could readily yield the intermediate **66**. Finally, the retro-Michael addition/pyridine extrusion cascade delivered the desired benzooxathiepine products. Moreover, the catalytic asymmetric version of the (3 + 4) cycloaddition reaction was explored to construct atropisomeric styrenes (Scheme 15, bottom). A series of bifunctional organocatalysts (not shown) were screened, and the researchers found that asymmetric (3 + 4) cycloaddition proceeded smoothly in the presence of a hydroquinine-based thiourea **C1** (10 mol%; catalyst) in dichloromethane (DCM) at room temperature, allowing the formation of the chiral compound **63a′** with good yield (82%) with moderate stereoselectivity (67% *ee*).

### 2.3. Formal (4 + n) Cyclization

Due to the existence of the unique sulfur atom extrusion process, the exploitation of sulfur-based pyridinium and quinolinium 1,4-zwitterions goes well beyond the conventional pyridinium ylide and 1,5-dipole concept. It was found that they can be regarded as four atom synthons participating in formal [4 + n] cyclization, allowing the facile synthesis of five- and six-membered rings. Building upon the reaction mechanism, the dearomatization of the heteroarenium ring and the desulfuration reaction always could have been observed in disclosed reports.

#### 2.3.1. Formal (4 + 1) Cyclization

**Scheme 16:**
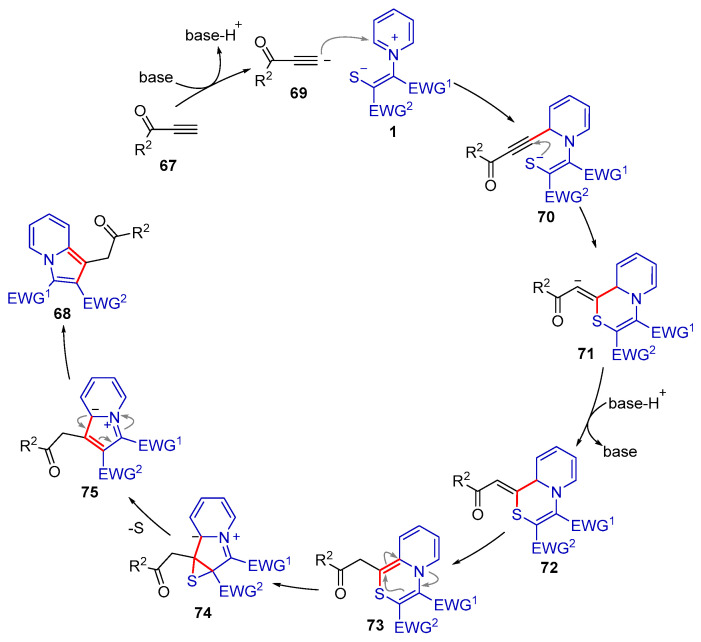
Possible reaction pathways in formal (4 + 1) cyclization reaction.

In 2020, Zhai et al. demonstrated the viability of a (4 + 1) cyclization reaction using pyridinium 1,4-zwitterions (Table 1) [76]. In the devised reaction, Et_3_N enabled the (4 + 1) cyclization of pyridinium 1,4-zwitterions **1** and propiolic acid derivatives **67** in DCM at 30 °C to furnish various indolizines **68** in yields ranging from 15% to 75%. According to the authors, the reaction mechanism involved the nucleophilic attack of an acetylide anion **69** on the pyridinium 1,4-zwitterion **1**, leading to the 1,2-dearomatization of the pyridine group and the formation of the intermediate **70**. An intramolecular S-Michael addition/protonation process gave birth to intermediate **72**, which underwent double bond isomerization to form the key intermediate **73**. A nitrogen-triggered intramolecular ring-contraction reaction produced intermediate **74**, which underwent a spontaneous sulfur atom extrusion process to yield intermediate **75**. Finally, the aromatization of intermediate **75** yielded the desired indolizine derivatives (Scheme 16).

**Scheme 17:**
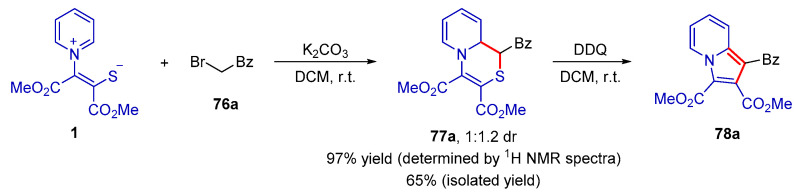
Reaction modes associated with pyridinium 1,4-zwitterions and α-functionalized bromoalkanes.

**Scheme 18:**
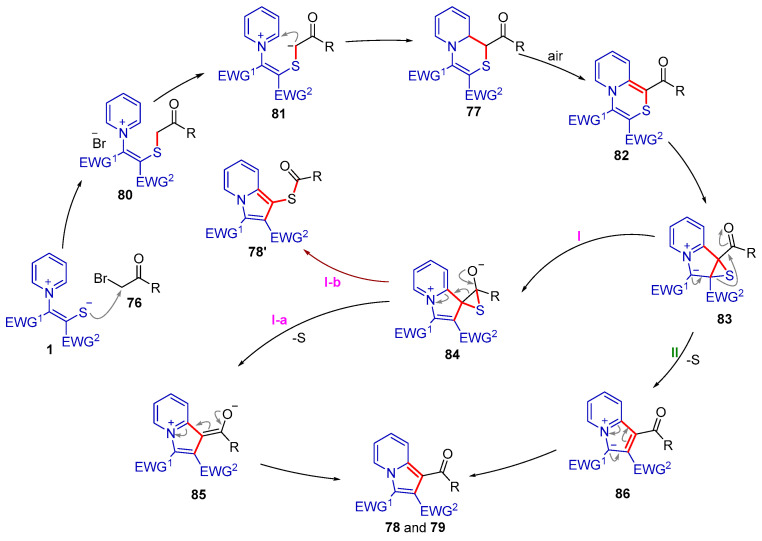
Proposed pathway for the formal (4 + 1) cyclization reaction of pyridinium 1,4-zwitterions and bromoalkanes.

Zhai et al. developed a one-pot formal (4 + 1) cyclization reaction involving sulfur-based pyridinium 1,4-zwitterions **1** and *α*-functionalized bromoalkanes **76** [77]. Initially, they achieved a (5 + 1) cyclization to access pyridothiazine **77a** with poor diastereoselectivity, and the inherent instability of pyridothiazine resulted in a low, isolated yield. Fortunately, they solved the issue using 2,3-dichloro-5,6-dicyano-1,4-benzoquinone (DDQ) as an oxidant and successfully carried out the oxidation of pyridothiazine (Scheme 17). Further investigation indicated that a two-step, one-pot formal (4 + 1) cyclization could be achieved, resulting in the formation of indolizines with acceptable results (Table 2). They reported that substituents had an obvious influence on the course of the reaction. The authors proposed a mechanism for the formation of pyridothiazine and indolizines (Scheme 18). The *S*-nucleophilic substitution of pyridinium 1,4-zwitterion **1** with bromoalkene **76** yielded an intermediate **80**. Then, an intramolecular nucleophilic addition performed on pyridinium delivered pyridothiazine **77**, which subsequently underwent oxidation to afford an intermediate **82**. An intramolecular Michael reaction resulted in the formation of a key intermediate **83**. The intermediate **83** could react following two plausible pathways under the influence of the substituent. The first pathway involved the formation of a spiro-thiirane **84**. A sequential desulfuration reaction/tautomerization reaction afforded indolizine **78** as the major product. A ring-opening reaction of the intermediate **84** delivered an *S*-(indolizin-1-yl)benzothioate **78′** as a byproduct. In the second pathway, the intermediate **83** directly underwent desulfuration and tautomerization to yield indolizine **79** as the sole product.

#### 2.3.2. Formal (4 + 2) Cyclization

**Scheme 19:**
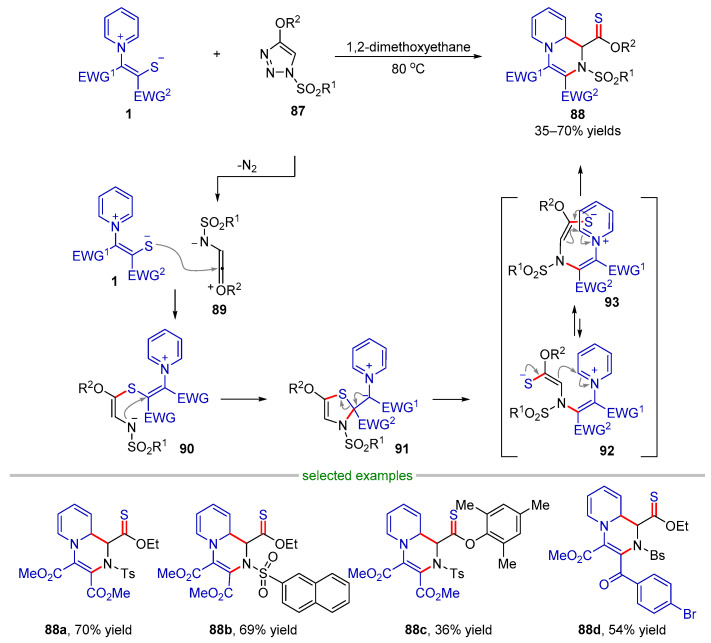
The formal (4 + 2) cyclization reaction between pyridinium 1,4-zwitterions and 1-sulfonyl-1,2,3-triazoles.

There is only one example of using pyridinium 1,4-zwitterions as four-atom synthons in formal (4 + 2) cyclization for the synthesis of six-membered ring compounds. In 2021, Li et al. achieved a (4 + 2) cyclization between 1-sulfonyl-1,2,3-triazoles **87** and pyridinium 1,4-zwitterions **1** through an addition/elimination process, accessing pyrido[1,2-*a*]pyrazine derivatives **88**. The products were formed in yields of up to 70% (Scheme 19) [57]. The authors proposed a mechanism to explain the observed results. Under thermal conditions, a key intermediate **89** was generated from the 1,2,3-triazole **87** with the release of nitrogen. Following this, a sequential *S*-nucleophilic addition/*N*-Michael reaction proceeded to yield a thiazole intermediate **91**. A retro-*S*-Michael reaction resulted in the formation of intermediates **92** and **93**, which underwent an intramolecular nucleophilic attack from the carbon atom to access the final product, **88**. The key features of the developed procedure are that it is catalyst-free and easy to operate.

### 2.4. Formal (5 + n) Cyclization

Formal (5 + n) cyclization of sulfur-based pyridinium and quinolinium 1,4-zwitterions has been proven to be a straightforward and powerful tactic for the construction of *N*/*S*-containing polyheterocyclic skeletons, but this has not been studied in detail. In this section, the newly reported (5 + 1) and (5 + 2) cyclization processes will be presented and discussed in detail.

#### 2.4.1. Formal (5 + 1) Cyclization

**Scheme 20:**
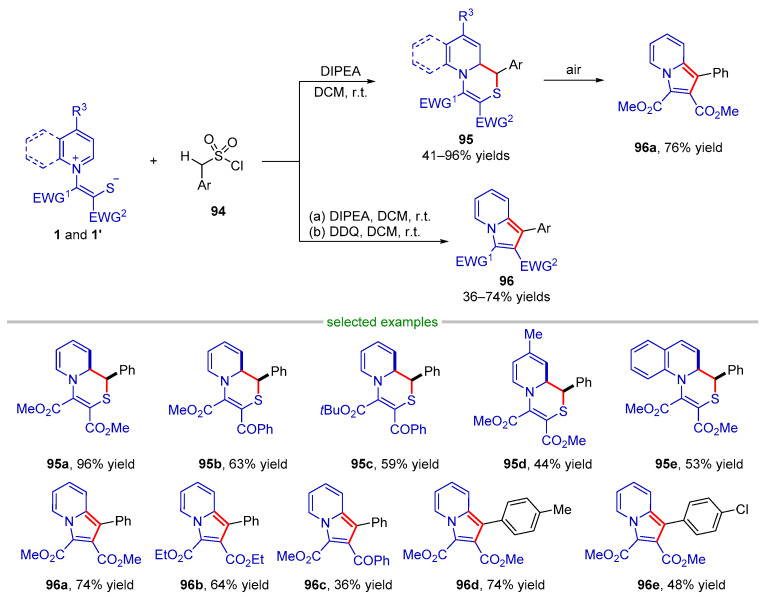
Formal (5 + 1) cyclization reaction of sulfur-based pyridinium 1,4-zwitterions with arylmethanesulfonyl chlorides.

**Scheme 21:**
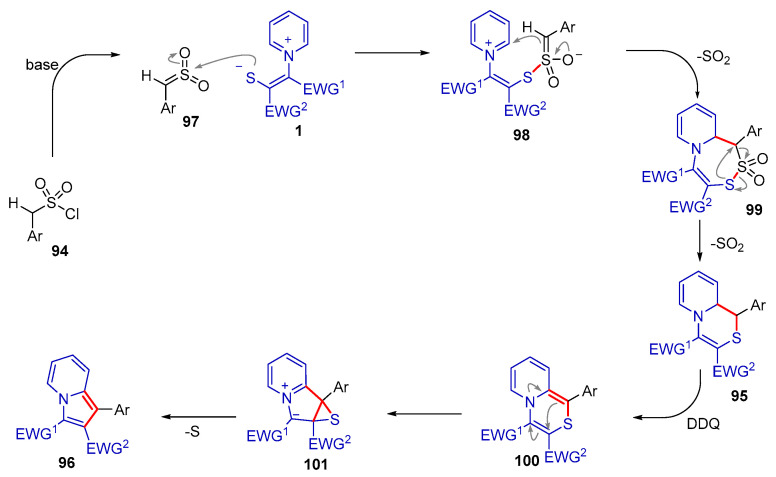
Mechanism for formal (5 + 1) cyclization reaction of sulfur-based pyridinium 1,4-zwitterions and arylmethanesulfonyl chlorides.

In 2020, Zhai, Cheng, et al. reported the (5 + 1) cyclization of pyridinium and quinolinium 1,4-zwitterions with arylmethanesulfonyl chlorides **94** using *N*,*N*-diisopropylethylamine (DIPEA) as a promoter (Scheme 20) [55]. The reaction proceeded smoothly, yielding the corresponding dihydropyrido[2,1-*c*][1,4]thiazines **95** in generally high yields (up to 96%). The authors discovered that due to the inherent instability of product **95**, an intramolecular ring-contraction reaction could occur in the presence of an oxidant. A preliminary study showed that using DDQ as the oxidant could achieve the transformation in a short time. As a result, a two-step one-pot conversion reaction of 1,4-zwitterions and arylmethanesulfonyl chlorides **94** was performed, furnishing indolizines **96** in good yields (36–74%) through a step-wise ((5 + 2) − 1) pathway. The mechanism is shown in Scheme 21. The reaction started with the in-situ generation of sulfene **97**, which was attacked by the sulfur anion of pyridinium 1,4-zwitterion **1** to produce intermediate **98**. The *α*-carbon of the sulfonyl group was then added to the pyridine ring, and this was followed by the transformation of 1,2,5-dithiazepane **99** to product **95** via an SO_2_ extrusion reaction. Product **95** underwent oxidation/ring contraction to yield indolizines **96** in the presence of DDQ.

**Scheme 22:**
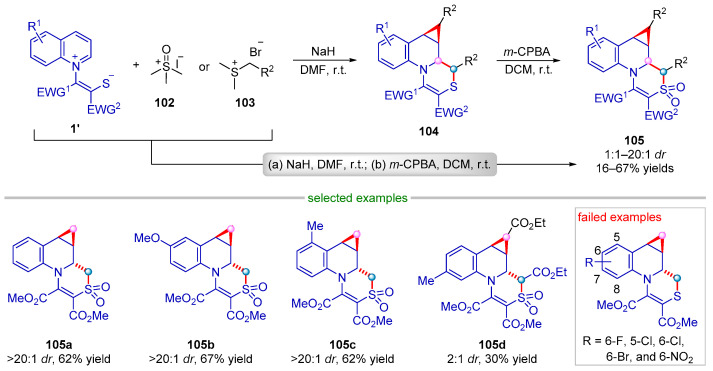
Cascade (2 + 1)/(5 + 1) cyclization of sulfur-based quinolinium 1,4-zwitterions with sulfur ylide salts.

**Scheme 23:**
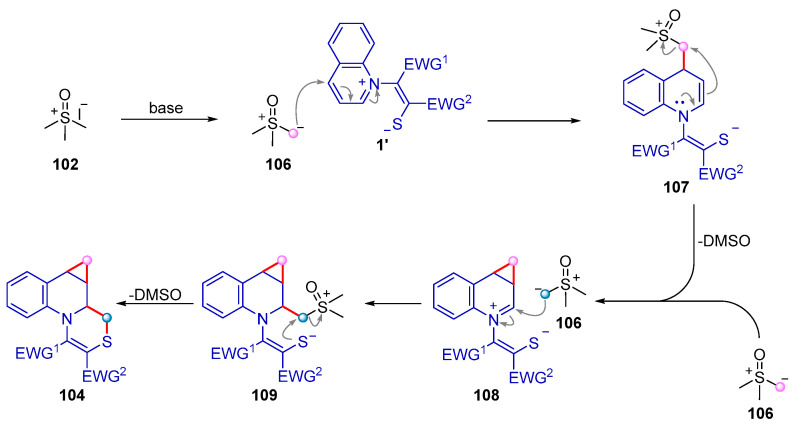
Reaction mechanism associated with the cascade (2 + 1)/(5 + 1) cyclization reaction.

In 2021, Zhang, Jin, et al. reported a metal-free cascade (2 + 1)/(5 + 1) cyclization reaction involving quinolinium 1,4-zwitterions **1′** and sulfur ylide salts (**102** and **103**) for the synthesis of cyclopropa[*c*][1,4]thiazino-[4,3-*a*]quinolines **104** with excellent diastereoselectivity (Scheme 22) [78]. To overcome the difficulties in separation and purification, they explored the process of selective oxidation of product **104** and found that a one-pot step-wise reaction smoothly produced sulfone analogs **105** in excellent isolated yields with perfect diastereoselectivities. The scope of the reaction was investigated, but the protocol was not applied to quinolinium 1,4-zwitterions **1′** that bear the electron-deficient groups at the fifth or sixth position of the quinolinium ring (Scheme 22, bottom). The authors proposed the mechanism with sulfur ylide salt **102** as an example. They hypothesized that the reaction involved the in-situ formation of sulfoxonium ylide **106**, which underwent nucleophilic attack on the quinolinium zwitterion to form intermediate **107**. This was followed by an intramolecular nucleophilic substitution reaction that yielded **108**. The (5 + 1) cyclization reaction between intermediate **108** and another sulfoxonium ylide **106** gave rise to the final product **104**. DMSO was released during the process (Scheme 23).

**Scheme 24:**
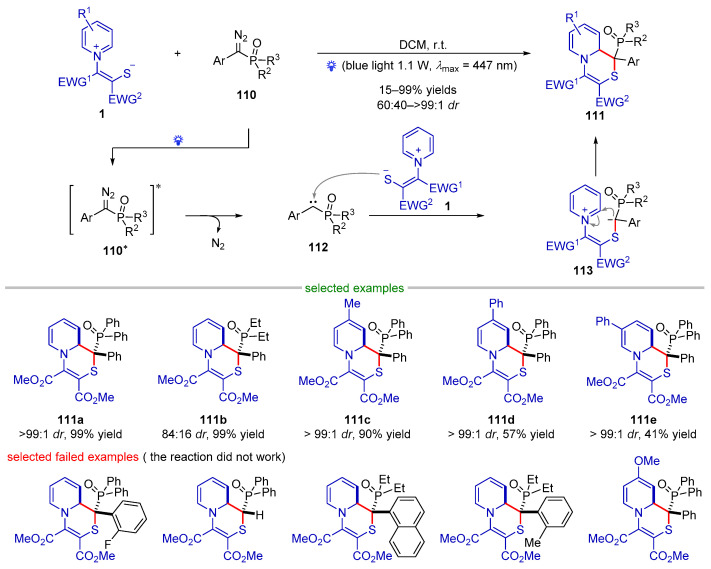
Visible-light-induced (5 + 1) cyclization reaction involving pyridinium 1,4-zwitterions and phosphoryl diazo compound.

Visible-light photocatalysis is an environmentally friendly strategy that has been used for the synthesis of various organic compounds over the past decade [79,80,81,82,83,84]. In 2022, Xu et al. made a significant breakthrough by introducing the first blue-light-induced annulation of pyridinium 1,4-zwitterions (Scheme 24) [85]. In their developed methodology, the phosphoryl diazo compound **110** was selected as the precursor of an electron-deficient carbene, and the compound was excited under conditions of blue-light irradiation to produce carbene intermediate **112**, which then reacted with pyridinium 1,4-zwitterion **1** through a (5 + 1) cyclization reaction. The reactions resulted in the production of phosphoryl-1,9*a*-dihydropyrido[2,1-*c*][1,4]thiazine derivatives **111** in generally good yields (15–99%) and diastereomeric ratios (60:40–>99:1 *dr*). It is worth noting that steric hindrance and electronic effects significantly impacted the reactivity of the molecules, and the developed method could not be applied to some substrates.

#### 2.4.2. Formal (5 + 2) Cyclization

**Scheme 25:**
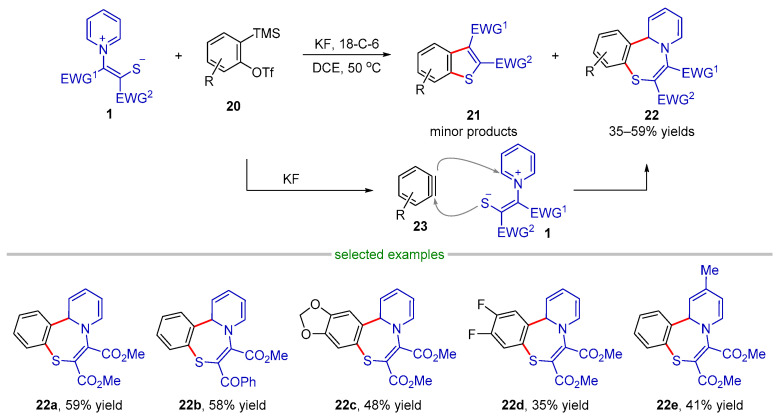
The (5 + 2) cyclization reaction of sulfur-based pyridinium 1,4-zwitterions with in situ-formed benzyne.

In 2020, Zhai et al. reported one of the only two instances of (5 + 2) cyclization of pyridinium 1,4-zwitterions to construct seven-membered sulfur-containing heterocyclic rings (Scheme 25) [54]. In this example, the in situ-generated benzyne **23** underwent 1,5-dipolar cycloaddition with pyridinium 1,4-zwitterions **1**, resulting in the formation of benzopyridothiazepines **22** as the major product. However, due to regioselectivity, a (3 + 2) cascade cyclization reaction also produced benzo[*b*]thiophenes **21** as a side product.

**Scheme 26:**
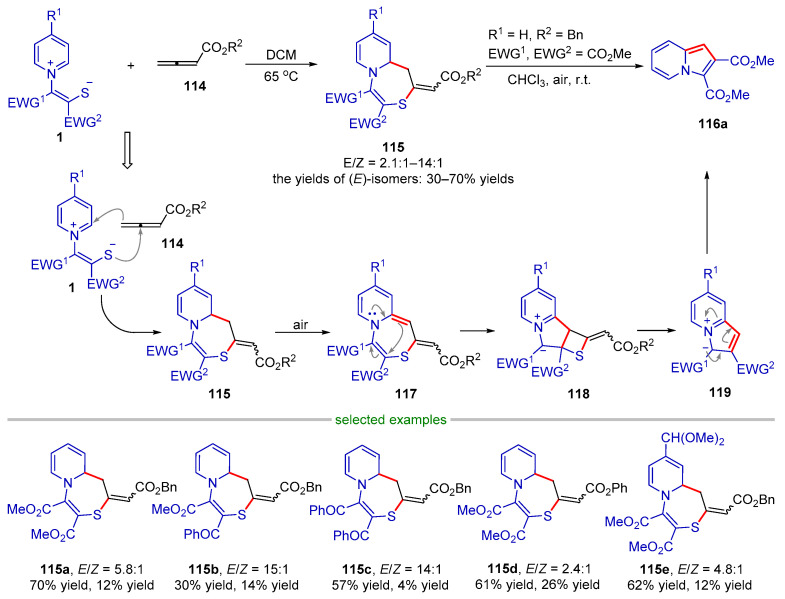
**The** (5 + 2) cyclization reaction of sulfur-based pyridinium 1,4-zwitterions with activated allenes.

Another example of a (5 + 2) cyclization is the synthesis of pyridothiazepines **115** via the reaction between pyridinium 1,4-zwitterions **1** and activated allenes **114** (Scheme 26) [86]. The corresponding pyridothiazepine derivatives **115** were obtained in good yields with acceptable *Z*/*E* configuration when the reaction was conducted at 65 °C in DCM. A ring-contraction reaction of **115a** could also be achieved in an air atmosphere, furnishing indolizine **116a** as the final product. The authors proposed a possible mechanism for the (5 + 2) cyclization and subsequent ring-contraction reaction. First, a highly regioselective (5 + 2) cyclization resulted in the formation of pyridothiazepine **115**. Due to its instability, the aerobic oxidation of pyridothiazepine **115** yielded a conjugated double bond, and this was followed by an intramolecular nucleophilic addition that yielded intermediate **118**. An extrusion reaction of intermediate **118** produced **119**, which underwent an efficient isomerization process to synthesize indolizine **116**. The authors noted that the electronic nature of the R^1^ group could dictate the pathway of the reaction.

### 2.5. Multistep Cascade Cyclization

**Scheme 27:**
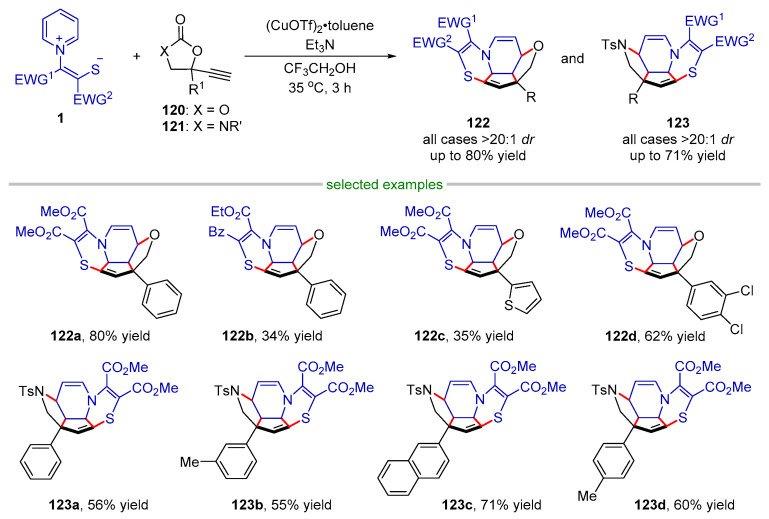
Cu(I)-catalyzed decarboxylative multistep cascade cyclization of pyridinium 1,4-zwitterions and propargylic cyclic carbonates/carbamates.

**Scheme 28:**
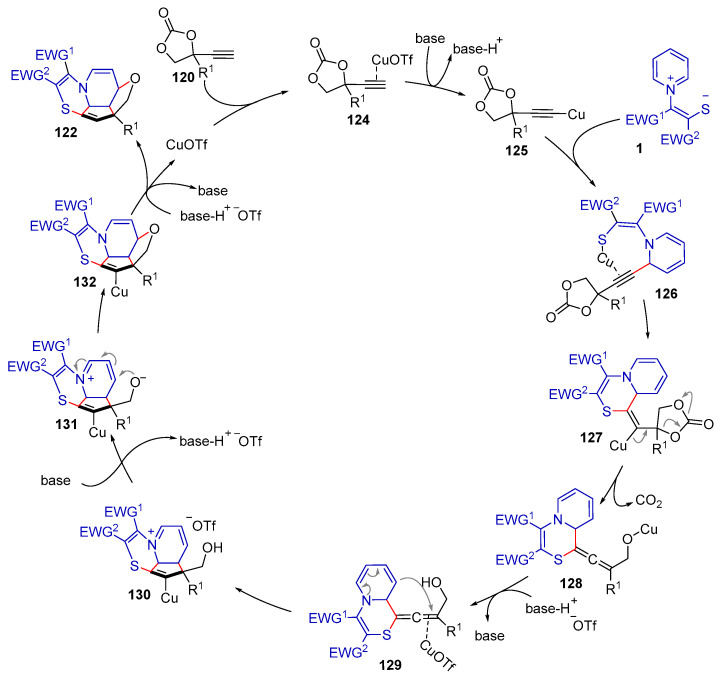
Possible reaction mechanism for copper-catalyzed decarboxylative multistep cascade cyclization reaction.

The transition-metal-catalyzed decarboxylative cyclization of alkyne-substituted carbonates has emerged as an effective strategy for the construction of various heterocycles [87,88,89,90]. In 2022, Yuan et al. described a copper-catalyzed decarboxylative cascade cyclization of propargylic cyclic carbonates **120**/carbamates **121** with pyridinium 1,4-zwitterions **1** (Scheme 27) [91]. (CuOTf)_2_·toluene catalyzed the cascade cyclization in the presence of Et_3_N, and the reaction afforded fused polyheterocycles **122** and **123** in comparable yields with excellent diastereoselectivities. The reaction proceeded under mild reaction conditions and four new bonds (two C−C, one C−O/N, and one C−S) were formed efficiently in a single step. The mechanism of the reaction was elucidated, as shown in Scheme 28. At first, the copper catalyst activated the alkyne fragment of **120** to form the π–alkyne copper intermediate **124**, and this was followed by a deprotonation reaction that resulted in the generation of the copper–acetylide intermediate **125**. Subsequently, the nucleophilic addition of intermediate **125** to pyridinium 1,4-zwitterion **1** led to the 1,2-dearomatization of the pyridine ring, resulting in the formation of the intermediate **126**, which underwent a 6-*exo*-cyclization reaction to yield intermediate **127**. The sequential carbonate ring opening and decarboxylation of **127** resulted in the formation of the heterocyclic tetrasubstituted allenolated copper species **128**. Thereafter, intermediate **129,** formed following the protonation of **128**, underwent an intramolecular cyclization reaction to furnish the tricyclic intermediate **130**. This was followed by an intramolecular oxa-conjugate addition reaction to promote the formation of the tetracyclic vinylcopper intermediate **132**. Finally, the protodemetalation of **132** delivered the target product **122**.

## 3. Nitrogen-Based Pyridinium and Quinolinium 1,4-Zwitterions

Nitrogen-based pyridinium 1,4-zwitterions **2** were first reported by Yoo et al. in 2014 (Table 3) [49]. It was found that conducting a Rh_2_(esp)_2_-catalyzed reaction between 1-sulfonyl-1,2,3-triazole **133a** and 2-phenylpyridine **134a** could afford isolable pyridinium 1,4-zwitterion **2a**. A wide range of isolable pyridinium 1,4-zwitterions **2** was successfully synthesized in excellent yields when optimized reaction conditions were used to conduct the studies. The use of nitrogen-based pyridinium 1,4-zwitterions has increased over the years as the compounds are highly reactive and contain multiple reaction sites. The reactions are centered upon formal (3 + 2) cyclization, (5 + n) cyclization, cascade dearomative (2 + n) cycloaddition/intramolecular cyclization, and 1,4-dearomative ring expansion/intramolecular cyclization reactions. These reactions have been discussed in the above sequence and in the following sections.

### 3.1. Formal (3 + 2) Cyclization

**Scheme 29:**
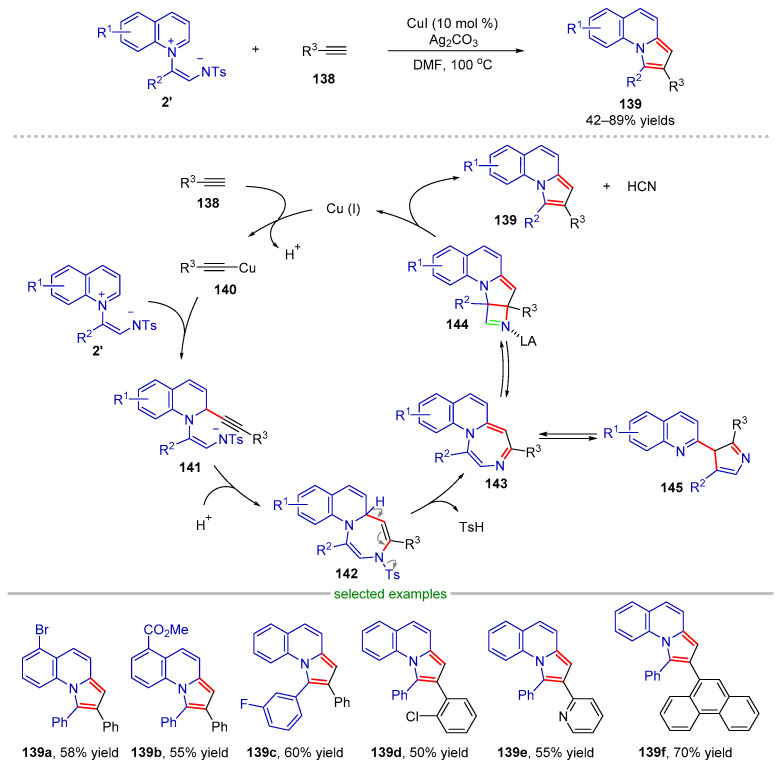
Cu(I)-catalyzed formal (3 + 2) cyclization between quinolinium 1,4-zwitterions and terminal alkynes.

Studies on (3 + 2) cyclization reactions involving nitrogen-based pyridinium or quinolinium 1,4-zwitterions have been almost completely absent since 2014. The sole example was reported by Yoo et al. in 2021. As shown in Scheme 29, Cu(I) was selected as the catalyst to react with terminal alkyne **138** to generate copper acetylide **140**. Copper acetylide **140** regioselectively attacked the 2-position of quinolinium to achieve 1,2-dearomatization and yield intermediate **141**. Intermediate **141** could convert to 1,4-diazepine intermediate **142** via the process of 7-endo-dig cyclization. This was followed by detosylation to yield **143**. The unstable 8π-electron of **143** participated in the reaction and allowed the sequential 4π-electro-cyclization reaction to proceed smoothly, affording intermediate **144**. The retro-(2 + 2) cycloaddition reaction resulted in the release of HCN gas, delivering the desired pyrrolo[1,2-*a*]quinoline **139** in the presence of Ag_2_CO_3_ [92]. The developed (3 + 2) cyclization reaction worked well in moderate-to-good yields (42–89%). Of note, the stable valence tautomer **145** was also formed under special conditions, and this could be attributed to the dynamic equilibrium between **143**, **144**, and **145**. The silver, salt-mediated HCN gas release process functioned as a driving force to facilitate the formation of the final product **139**.

### 3.2. (5 + n) Cyclization

Nitrogen-based pyridinium and quinolinium 1,4-zwitterions that function as 1,5-dipoles could be used as five-atom synthons in (5 + n) cyclization reactions to access six-, seven-, and eight-membered dinitrogen-fused heterocycles.

#### 3.2.1. (5 + 1) Cyclization

**Scheme 30:**
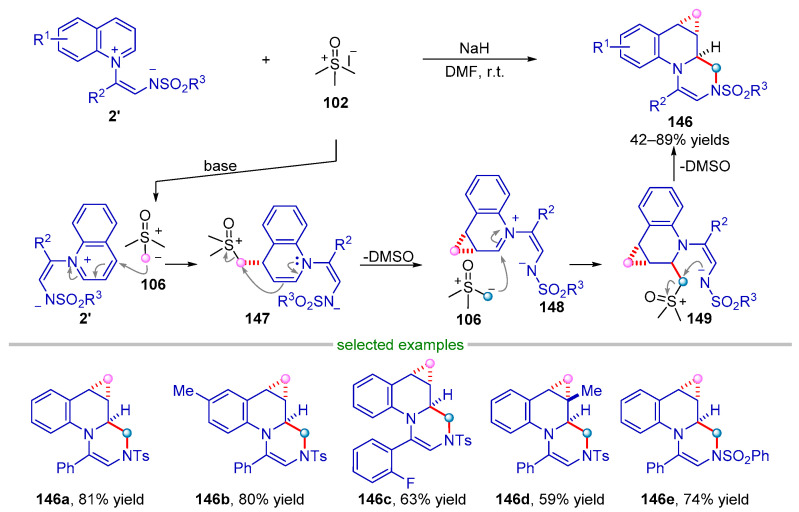
Cascade (2 + 1)/(5 + 1) cycloaddition reaction of nitrogen-based quinolinium 1,4-zwitterions with trimethylsulfoxonium iodide.

The cascade 1,4-dearomative (2 + 1) and (5 + 1) cycloaddition reactions of quinolinium 1,4-zwitterions **2′** with trimethylsulfoxonium iodide **102** were studied by Yoo et al. in 2020 (Scheme 30) [93]. The reaction proceeded smoothly in the presence of NaH in DMF to give rise to cyclopropane-fused pyrazino[1,2-*a*]quinolines **146** in good yields, and high levels of diastereocontrol could be achieved under these conditions. Based on the control experiments, the authors concluded that the (2 + 1) cycloaddition reaction of 1,4-zwitterion **2′** and sulfoxonium ylide **106** led to the generation of the key intermediate **148**. This reaction was followed by the (5 + 1) cyclization reaction in the presence of another sulfoxonium ylide **106**, yielding the corresponding product **146**.

**Scheme 31:**
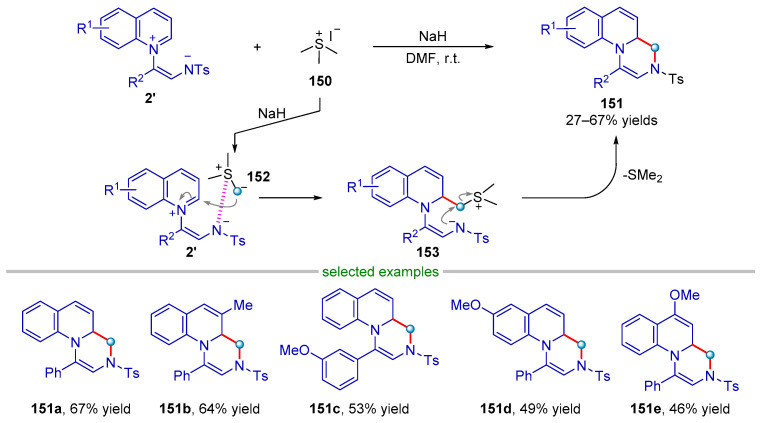
Formal (5 + 1) cycloaddition of nitrogen-based quinolinium 1,4-zwitterions and trimethylsulfoxonium iodide.

The (5 + 1) cycloaddition reaction with quinolinium 1,4-zwitterions **2′** and sulfonium ylide salt **150** was conducted by Yoo et al. in 2021 following the success of cascade 1,4-dearomative (2 + 1) and (5 + 1) cycloaddition reactions (Scheme 31) [94]. When the sulfonium ylide **152** was used as a simple nucleophile, the quinolinium ring of the 1,4-zwitterion underwent a highly regioselective 1,2-dearomative addition reaction to deliver intermediate **153**. Subsequently, the classical nucleophilic substitution reaction involving intermediate **153** afforded cycloadduct **151** in moderate-to-good yields (27–67%). In mechanistic analysis, the authors were much more likely to conclude that the chelation between the nitrogen anion of the 1,4-zwitterion and the sulfur cation of sulfonium ylide resulted in excellent regioselectivity (as seen in **152**).

**Scheme 32:**
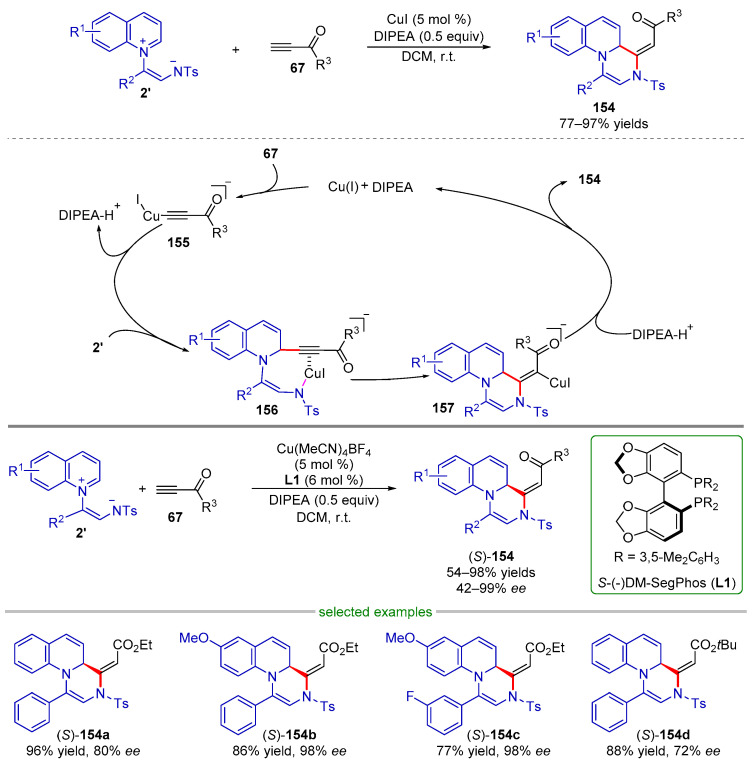
Cu(I)-catalyzed (5 + 1) cycloaddition involving nitrogen-based quinolinium 1,4-zwitterions and activated terminal alkynes.

Activated terminal alkynes **67** functioned as one-carbon synthons in the (5 + 1) cyclization of quinolinium 1,4-zwitterions **2′** in 2020 (Scheme 32) [95]. The reaction began with the formation of nucleophilic copper acetylide **155**, which then attacked the C2 position of the quinolinium ring to form intermediate **156**. Subsequently, intermediate **156** underwent a 6-exo-cyclization reaction, resulting in the formation of the heterocyclic intermediate **157**. Finally, protonation of **157** resulted in the formation of pyrazino[1,2-*a*]quinoline compound **154** in good yields with excellent regioselectivities. Density functional theory (DFT) calculations indicated that the binding of the copper catalyst to the amide-nitrogen was responsible for the observed excellent regioselectivity (as seen in **156**). Additionally, an enantioselective version of the (5 + 1) cyclization reaction between quinolinium 1,4-zwitterions and activated terminal alkynes was also conducted (as shown at the bottom of Scheme 32). The chiral pyrazino[1,2-*a*]quinoline derivatives (*S*)-**154** were produced in excellent yields (up to 98%) with high enantioselectivities (up to 99% *ee*) in the presence of Cu(MeCN)_4_BF_4_ and the *S*-(−)DM-SegPhos (**L1**) complex.

#### 3.2.2. (5 + 2) Cyclization

In 2014, Yoo et al. conducted the first (5 + 2) cyclization reaction for nitrogen-based pyridinium 1,4-zwitterions. As shown in Table 4, dimethyl acetylenedicarboxylates (DMADs) were used as reactants in the (5 + 2) cyclization process under thermal conditions. The 1,4-diazepine compounds **158** could be isolated in excellent yields (following Path a) [49]. Furthermore, a two-step, one-pot (5 + 2) cyclization reaction could also be performed at 120 °C to yield a wide range of desired products **158** in good yields (following Path b). Additionally, a four-component annulation reaction was also investigated and carried out successfully, producing 1,4-diazepines in acceptable yields (following Path c). The broad substrate scope, good tolerance of functional groups, and unique reaction pathways demonstrated the versatility of the developed methodology.

**Scheme 33:**
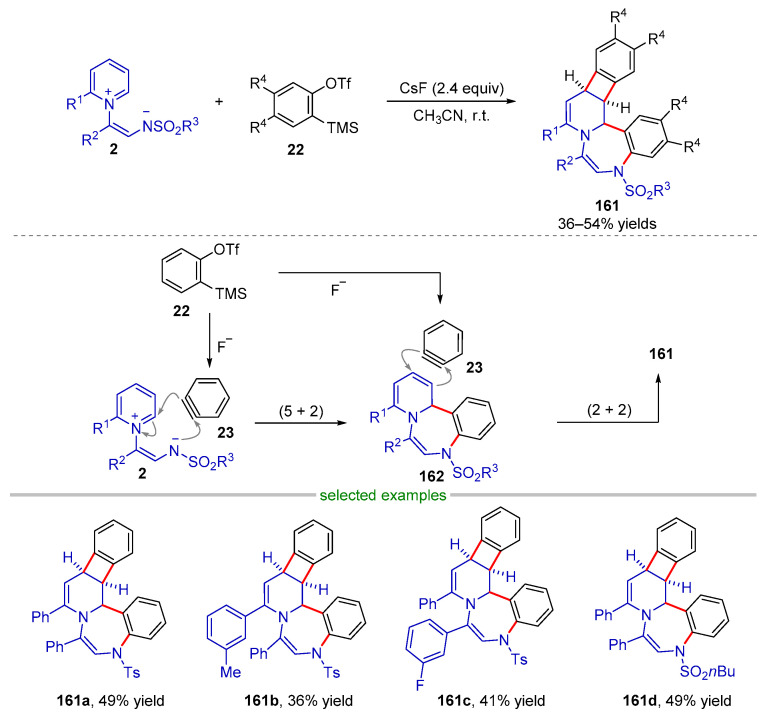
Metal-free cascade (5 + 2)/(2 + 2) cyclization between pyridinium 1,4-zwitterions and in situ-generated arynes.

A metal-free cascade (5 + 2)/(2 + 2) cyclization reaction between pyridinium 1,4-zwitterions **2** and in situ-generated arynes **23** was described in 2017 [96]. As illustrated in Scheme 33, fluoronium promoted the in-situ formation of arynes **23** from silylaryl triflate **22**, followed by a (5 + 2) cyclization reaction with pyridinium 1,4-zwitterion **2**, resulting in the formation of the 1,2-dearomative intermediate **162**. Finally, (2 + 2) cyclization of intermediate **162** with another molecule of benzyne **23** produced pentacyclic 1,4-benzodiazepine **161** in acceptable yields (36–54%). This reaction was characterized by the recovery of pyridinium 1,4-zwitterions, a broad substrate scope, and mild reaction conditions.

**Scheme 34:**
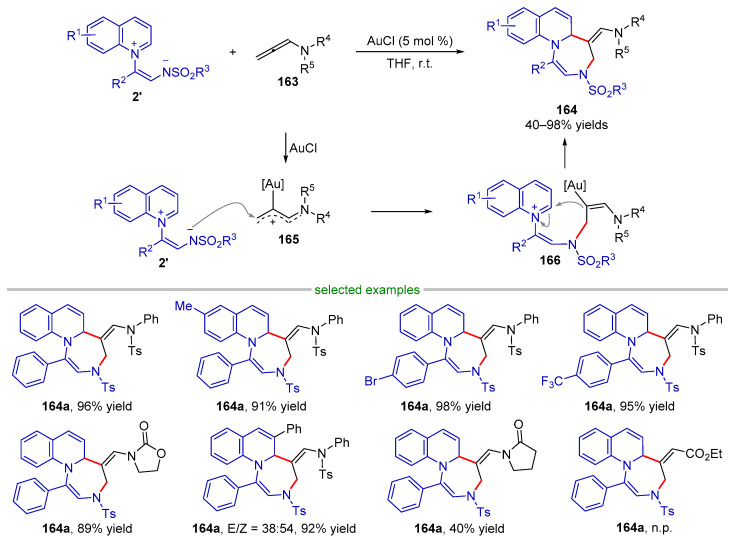
Au(I)-catalyzed (5 + 2) cyclization of nitrogen-based pyridinium 1,4-zwitterions and allenamides.

Gold(I)-catalyzed cyclization reactions are some of the most powerful and widely used synthetic methods for the synthesis of cyclic compounds [97,98,99]. In the context of a gold catalysis, the transformation of compounds based on the (5 + 2) cyclization of allenamides **163** and 1,4-zwitterions **2′** was reported in 2018 (Scheme 34) [100]. Quinolinium 1,4-zwitterions **2′** smoothly took part in the reaction and transformed into polycyclic 1,4-diazepines **164**. The maximum yield of the products was recorded to be 98%. For the reaction mechanism, in the presence of a gold catalyst, allenamide could convert into an Au-bound allylic cation **165**, which was attracted by the nitrogen anion of quinolinium 1,4-zwitterion to generate intermediate **166**. Finally, intramolecular 1,2-dearomative cyclization delivered the target compound **164**.

**Scheme 35:**
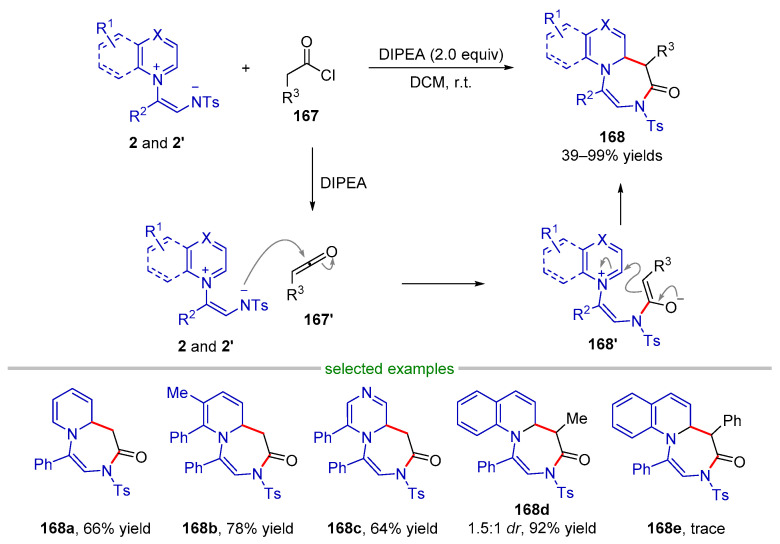
The DIPEA-promoted (5 + 2) cyclization reaction of nitrogen-based *N*-heteroarenium 1,4-zwitterions with in situ-generated ketenes.

In 2018, Yoo et al. prepared a series of *N*-heteroaromatic rings derived 1,4-zwitterions [101]. Nuclear independent chemical shift (NICS(0)) values and structural calculations revealed that the aromaticity of the heteroaromatic ring strongly influenced the stability of 1,4-zwitterions (not shown). In their report, the (5 + 2) cyclization of *N*-heteroarenium 1,4-zwitterions with acetyl chloride **167** was also carried out with DIPEA as the base. 1,5-diazepinone derivatives **168** could be synthesized in 39–99% yields (Scheme 35). The reaction between acetyl chloride **167** with DIPEA was conducted smoothly in situ-generated ketene **167′**, which was attracted the nitrogen anion of 1,4-zwitterions to give intermediate **168′**. Then, the final product 168 was delivered through the intramolecular 1,2-dearomative cyclization of intermediate **168′**. It is of note that the cyclization reaction provided the desired product when alkyl chloride was used under the current conditions. On the contrary, the developed (5 + 2) cyclization reaction could not be conducted with the in situ-formed aryl ketene.

#### 3.2.3. (5 + 3) Cyclization

**Scheme 36:**
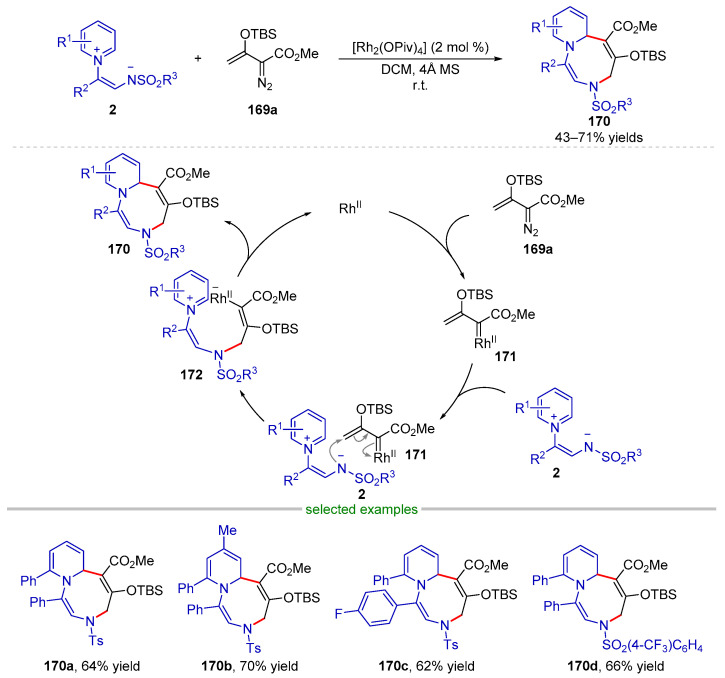
Rh(II)-catalyzed (5 + 3) cyclization involving nitrogen-based pyridinium 1,4-zwitterions and enol diazoacetates.

(5 + 3) cyclization is one of the most effective methods to construct eight-membered heterocycles [102,103,104,105,106,107]. However, it is challenging to conduct the asymmetric version of the reaction, and this problem needs to be addressed. Nitrogen-based pyridinium and quinolinium 1,4-zwitterions, as the most representative 1,5-dipoles, can be used to readily synthesize chiral eight-membered heterocycles. In 2015, Yoo et al. developed the Rh(II)-catalyzed (5 + 3) cyclization of pyridinium 1,4-zwitterions **2** and enol diazoacetates **169a** (Scheme 36) [108]. Modest yields of products **170** were observed (maximum yield: 71%). The mechanism consisted of three steps, as outlined in the middle of Scheme 36. The first step involved the reaction between enol diazoacetate and Rh(II), and this reaction yielded the Rh(II)-enolcarbene **171**. Next, Rh(II)-enolcarbene interacted with pyridinium 1,4-zwitterions to form intermediate **172**. Finally, intramolecular cyclization yielded the corresponding compound **170** while regenerating the active Rh(II) catalyst for the next cycle. Additionally, a chiral Rh(II) catalyst was used to promote the stereoselective (5 + 3) cyclization of pyridinium 1,4-zwitterion **2a** with TBS-protected enol diazoacetate **169a**. The stereoselective synthesis of chiral **170a** was achieved in a 60% yield with 90% *ee* when chiral Rh(II) catalyst **C4** was used to conduct the reaction (Table 5, entry 3).

**Scheme 37:**
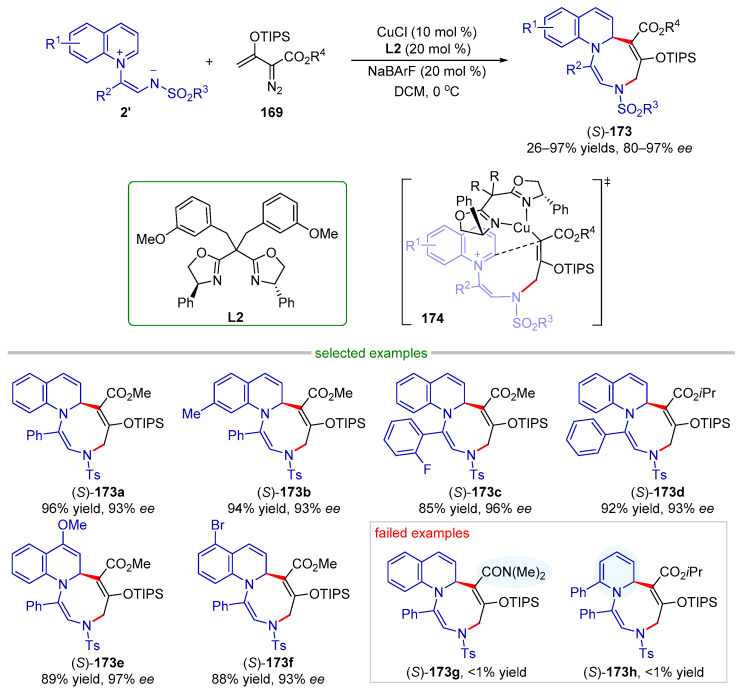
Cu(I)-catalyzed (5 + 3) cyclization between nitrogen-based quinolinium 1,4-zwitterions and enol diazoacetates.

Yoo et al. also described a stereoselective (5 + 3) cyclization between quinolinium 1,4-zwitterions **2′** and enol diazoacetates **169**, catalyzed by Cu(I), as shown in Scheme 37 [109]. The desired diazocine derivatives (*S*)-**173** could be synthesized in excellent yields (up to 97%) with perfect *ee* values (up to 97%) using a Cu(I)/bisoxazoline ligand L2 complex as a catalyst and a catalytic amount of NaBArF as an additive. The authors proposed that the non-coordinating anion of NaBArF enhanced the electrophilicity of the carbenoid intermediate during the reaction process. A transition state **174** was proposed, where the bisoxazoline ligand **L2** binds with the central Cu(I) to guide intramolecular 1,2-dearomative cyclization, thereby ensuring the observed enantioselectivities. It is worth noting that the reactions failed when either the enol diazoamide derivative or the pyridinium 1,4-zwitterion was used as a partner under the specified reaction conditions.

### 3.3. Cascade 1,4-Dearomative (2 + n) Cycloaddition/Intramolecular Cyclization

One remarkable feature of nitrogen-based pyridinium and quinolinium 1,4-zwitterions is their stability, which can be attributed to the aromaticity of the heteroarenium core. The selective dearomatization of the heteroarenium core intrigues many chemists. In 2018, Yoo et al. discovered that the charge delocalization property of the pyridinium zwitterion could be exploited for the selective 1,2- or 1,4-dearomatization of pyridinium [110]. Based on this discovery, various cascade 1,4-dearomative (2 + n) cycloaddition/intramolecular cyclization reactions have been developed in recent years.

#### 3.3.1. Cascade 1,4-Dearomative (2 + 1) Cycloaddition/Intramolecular Cyclization

**Scheme 38:**
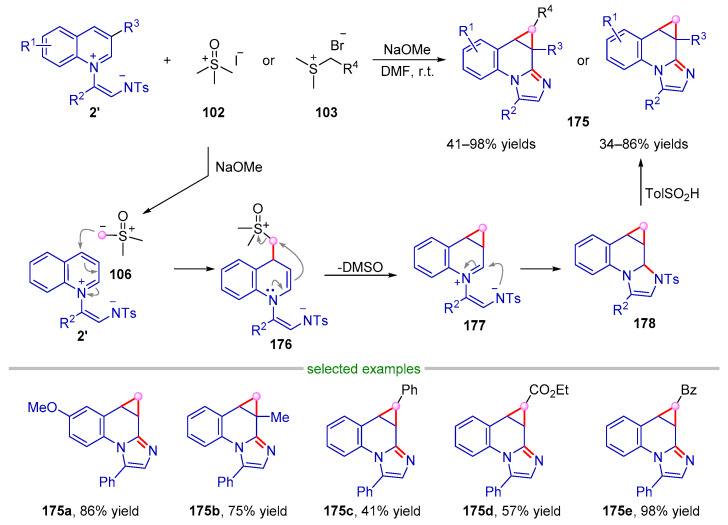
Cascade 1,4-dearomative (2 + 1) cycloaddition/intramolecular cyclization of nitrogen-based quinolinium 1,4-zwitterions and sulfur-based ylides.

The sole example of a cascade dearomative (2 + 1) cycloaddition/intramolecular cyclization was reported by Yoo in 2020 (Scheme 38) [93]. In this study, NaOMe (2.0 equiv. in DMF at 40 °C) was used as a base. Trimethylsulfoxonium iodide **102** or sulfonium ylide salt **103** was used to synthesize the corresponding product **175** in good-to-excellent yields (up to 98%). The mechanism (using **102** as an example) involved the nucleophilic addition of the in situ-generated sulfoxonium ylide **106** to the 4-position of quinolinium, resulting in 1,4-dearomatization and the formation of intermediate **176**. This was followed by cyclopropanation to form a cyclopropane ring and the smooth intramolecular cyclization of intermediate **177** to yield the tetrahydroimidazo[1,2-*a*]quinoline **178**. The rearomatization of **178** was achieved by extracting TolSO_2_H to give the final desired product **175**. Chiral benzyl sulfonium salts **179** could also be effectively used to conduct the reactions. Good, isolated yields, variable levels of diastereoselectivity, and excellent enantioselectivity were achieved when the reactions were conducted in the presence of NaH in acetonitrile at 40 °C (Table 6).

#### 3.3.2. Cascade 1,4-Dearomative (2 + 3) Cycloaddition/Intramolecular Cyclization

**Scheme 39:**
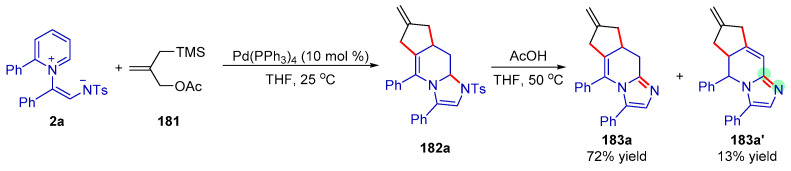
Pd(PPh_3_)_4_-catalyzed cascade reaction between TMM and pyridinium 1,4-zwitterion **2a**.

**Scheme 40:**
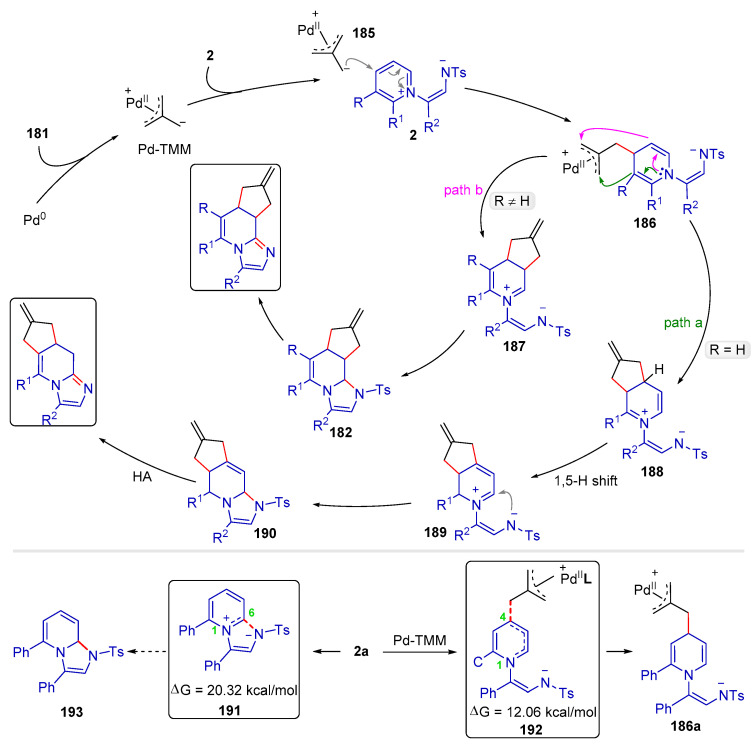
Plausible mechanisms for Pd-catalyzed cascade 1,4-dearomative (2 + 3) cycloaddition/intramolecular cyclization.

The 1,4-Dearomative (2 + 3) cycloaddition-triggered intramolecular cyclization of pyridinium 1,4-zwitterions was first disclosed in 2019 [111]. Pd(PPh_3_)_4_ was used to catalyze the dearomative (2 + 3) cycloaddition between trimethylenemethane (TMM) and pyridinium 1,4-zwitterion **2a**, resulting in the production of the unstable cycloadduct **182a**. Fortunately, the use of acidic additives promoted the elimination of sulfinic acid and the isomerization of the compound to furnish **183a** as the major product (Scheme 39). Evaluation of the substrate scope indicated that the efficiency and selectivity of the cycloadditions depended upon the nature of the substituent at the C3-position of pyridinium (Table 7). In the absence of substituents (R = H), compounds **183** were obtained in generally good yields. In contrast, C3-substituted pyridinium zwitterions were compatible with the developed strategy, but a totally different regioselectivity was observed. Compound **184** was smoothly generated in the absence of acetic acid. The authors hypothesized that the Pd(II)-TMM species **185** was initially generated when Pd(PPh_3_)_4_ reacted with TMM (Scheme 40, top). Following this, the Pd(II)-TMM species attracted pyridinium zwitterions to yield the key intermediate **186**. Next, the steric hindrance at the C3-position of pyridinium led to two pathways (Paths a and b) that could be followed to obtain the corresponding products. Furthermore, DFT calculations were conducted, and the computational results demonstrated that pyridinium 1,4-zwitterions were more likely to undergo C−C bond formation reactions, resulting in 1,4-dearomation and the formation of the intermediate **186** (Scheme 40, bottom).

**Scheme 41:**
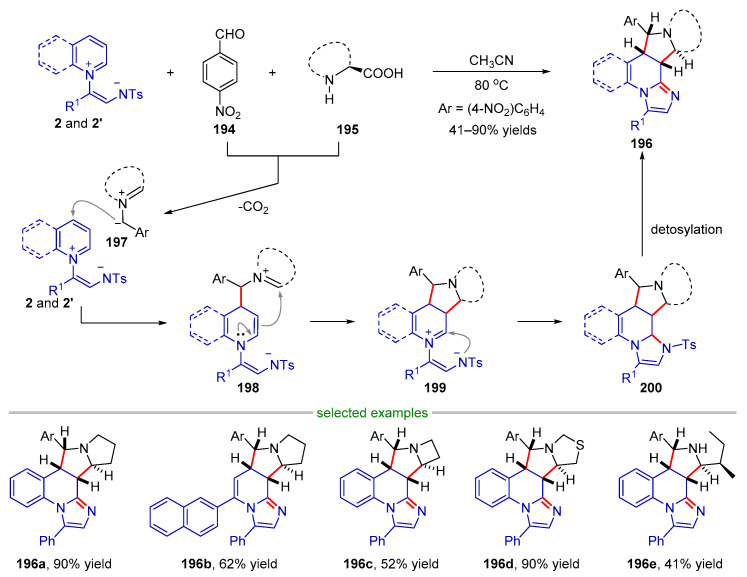
Multicomponent cascade dearomative (2 + 3) cycloaddition/intramolecular cyclization of nitrogen-based 1,4-zwitterions, aldehydes, and amino acids.

A strategy for multicomponent dipolar cycloaddition that involves the participation of in situ-formed azomethine ylides has been widely applied in the generation of nitrogen heterocyclic structures with a high level of functionality [112,113,114,115,116]. Yoo et al. have reported a catalyst-free multicomponent 1,3-dipolar cycloaddition/intramolecular cyclization reaction involving *N*-heteroarenium 1,4-zwitterions, aldehydes **194** and amino acids **195** (Scheme 41) [117]. The reaction was carried out in CH_3_CN at 80 °C and involved the decarboxylation of the aldehydes with amino acids to generate azomethine ylide **197**, which underwent a (2 + 3) cycloaddition reaction with pyridinium to give intermediate **199**. Intermediate **200** was then produced via intramolecular cyclization, and this was followed by the elimination of sulfinic acid, resulting in the formation of the desired product **196**. It is important to note that a strong electron-withdrawing group should be present at the *para*-position of the phenyl ring in aromatic aldehydes to achieve a high level of regioselectivity.

#### 3.3.3. Cascade 1,4-Dearomative (2 + 4) Cycloaddition/Intramolecular Cyclization

**Scheme 42:**
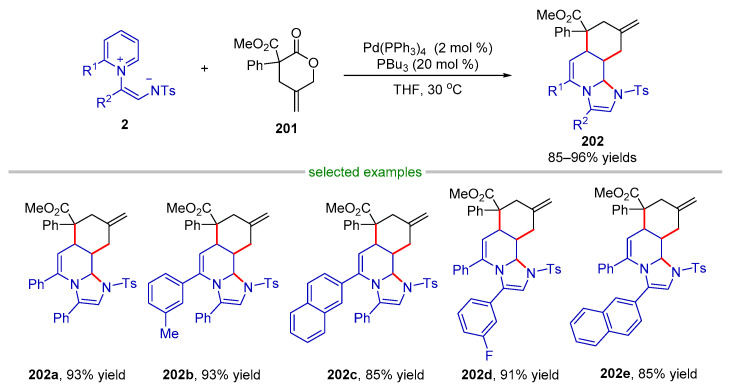
Pd-catalyzed cascade 1,4-dearomative (2 + 4) cycloaddition/intramolecular cyclization of nitrogen-based pyridinium 1,4-zwitterions with *γ*-methylidene-*δ*-valerolactone.

**Scheme 43:**
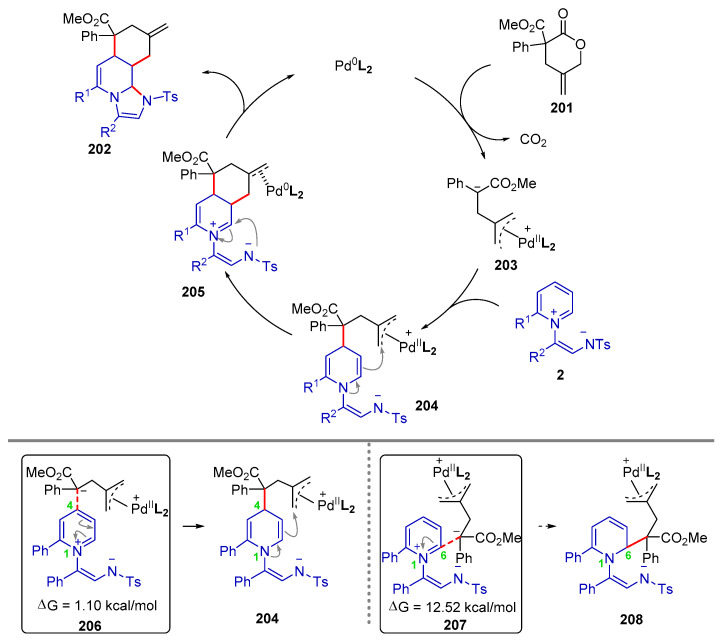
Proposed mechanism for Pd-catalyzed cascade 1,4-dearomative (2 + 4) cycloaddition/intramolecular cyclization.

The only example of cascade 1,4-dearomative (2 + 4) cycloaddition/intramolecular cyclization was reported by Yoo et al. in 2018 (Scheme 42) [110]. The decarboxylative cycloadditions of *γ*-methylidene-*δ*-valerolactone **201** with pyridinium 1,4-zwitterions **2** were catalyzed by Pd(PPh_3_)_4_ (2 mol%) and PBu_3_ (20 mol%) in tetrahydrofuran, producing various tetrahydroimidazo[1,2-*a*]pyridine derivatives **202** in excellent yields with perfect diastereoselectivities. The nucleophilic attack of the carbanion of Pd(II)-zwitterion species **203** on the C4 position of the pyridinium produced species **204**, which underwent intramolecular cyclization to form a six-membered ring. Finally, an intramolecular nucleophilic addition within intermediate **205** resulted in cyclization and produced the target compounds **202** (Scheme 43, top). DFT-based calculations indicated that the 1,4-dearomatization of the pyridinium moiety was thermodynamically favored (Scheme 43, bottom). The frontier molecular orbital (FMO) energy difference between the HOMO of the Pd(II)-zwitterion species **203** and the LUMO of the pyridinium zwitterion **2** was only 0.36 eV. This promoted efficient electronic coupling with a remarkably low barrier (not shown).

### 3.4. 1,4-Dearomative Ring Expansion/Intramolecular Cyclization

**Scheme 44:**
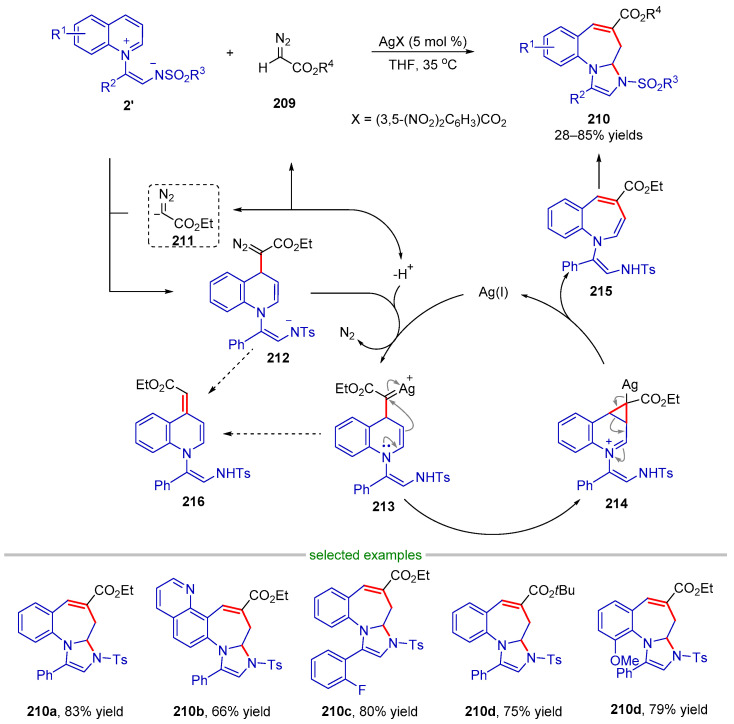
Ag(I)-catalyzed 1,4-dearomative ring expansion/intramolecular cyclization of quinolinium 1,4-zwitterions with diazoacetates.

Diazoacetate- and diazomethane-derived Grignard can achieve the 1,2-dearomative ring expansion of quinolinium to produce azepine derivatives [118]. In contrast, Yoo and Kim documented the 1,4-dearomative ring expansion of quinolinium using silver as a catalyst in 2021 (Scheme 44) [119]. They found that a broad range of functional groups were tolerated, and a high degree of regioselectivity, leading to the formation of multifused azepine derivatives **210** in good yields, could be achieved. Under optimized conditions, the in situ-generated diazoacetate anion **211** selectively attacked the C4 position of the quinolinium to effect 1,4-dearomatization and form intermediate **212**. The silver-carbenoid **213** was generated smoothly when a silver catalyst reacted with intermediate **212** via the release of nitrogen gas. The intramolecular cyclization of **213** resulted in the formation of cyclopropane intermediate **214**, followed by ring expansion to produce compound **215**. Finally, compound **215** converted into the desired azepine **210** following the process of intramolecular hydroamination. It is worth noting that a separable byproduct **216** was formed during the process, which might have been generated from intermediates **212** or **213**.

## 4. Summary and Outlook

In this review, we have summarized recent progress in the application of pyridinium and quinolinium 1,4-zwitterions for the efficient synthesis of heterocycles. The reported pyridinium and quinolinium 1,4-zwitterions can be classified into sulfur-based and nitrogen-based 1,4-zwitterions according to the types of anions. As to the study of sulfur-based 1,4-zwitterions, the known cyclization reactions include (2 + 3), (3 + n), (4 + n), (5 + n), and multistep cascade cyclization. With respect to nitrogen-based 1,4-zwitterions, different types of cyclization, such as (3 + 2) cyclization, (5 + n) cyclization, cascade 1,4-dearomative cycloaddition/intramolecular cyclization, and 1,4-dearomative ring expansion/intramolecular cyclization have been reported. The disclosed strategies have allowed the synthesis of a wide range of structurally diverse cyclic compounds, ranging from three- to eight-membered rings. However, there is still much room for improvement in this field. For example, the 1,4-dearomatization of sulfur-based, 1,4-zwitterion-triggered cyclization is limited, and only one report has been reported to date. Additionally, the use of nitrogen-based 1,4-zwitterions as pyridinium ylide-type synthons for the construction of nitrogen-containing heterocyclic compounds has not been reported to date. Some progress on the stereoselective reaction involving 1,4-zwitterions has also been made. However, the authors firmly believe that the exploration of asymmetric transformation is always worth pursuing. Additionally, photochemical catalysis is worth exploring in the field of transformations involving pyridinium and quinolinium 1,4-zwitterions.

We believe that this review will provide a useful reference for synthetic chemists who are interested in this area of work. The authors expect to see more progress and advances in the applications and scope of pyridinium and quinolinium 1,4-zwitterions in the near future. The authors also would like to apologize in advance for any unintentional omission of any literature report.

## Figures and Tables

**Table 1:** Formal (4 + 1) cyclization reaction of pyridinium 1,4-zwitterions with propiolic acid derivatives.

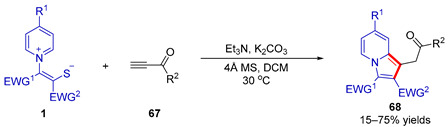					
Entry	R^1^	R^1^	EWG^1^	EWG^2^	The Yield of 68 (%)
1	H	OEt	COOMe	COOMe	**68a**/64
2	H	Bn	COOMe	COOMe	**68b**/63
3	H	OMe	COO*i*Pr	COO*i*Pr	**68c**/63
4	CH(OMe)_2_	OMe	COOMe	COOMe	**68d**/75
5	H	OPh	COOMe	COOMe	**68e**/n.d.
6	H	OMe	PhCO	PhCO	**68f**/trace

**Table 2:** One-pot formal (4 + 1) cyclization reaction between pyridinium 1,4-zwitterions and bromoalkanes.

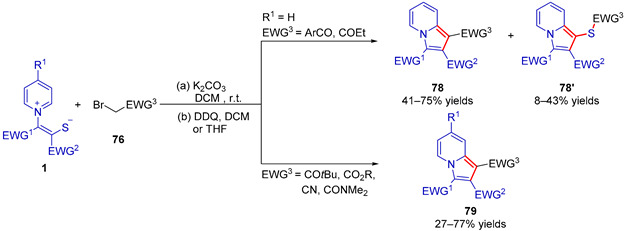						
Entry	R^1^	EWG^1^	EWG^2^	EWG^3^	Yield (%)	
1	H	COOMe	COOMe	C_6_H_5_CO	**78a**/73	**78a’**/26
2	H	COOMe	COOMe	2-furylCO	**78b**/46	**78b’**/8
3	H	COOMe	COOMe	COEt	**78c**/65	**78c’**/25
4	H	COOMe	COOMe	COOMe	**79a**/63	
5	H	COOMe	CO(4-BrC_6_H_4_)	COOBn	**79b**/62	
6	CH(OMe)_2_	COOMe	COOMe	COOBn	**79c**/77	

**Table 3:** Reactions for the synthesis of nitrogen-based pyridinium 1,4-zwitterions.

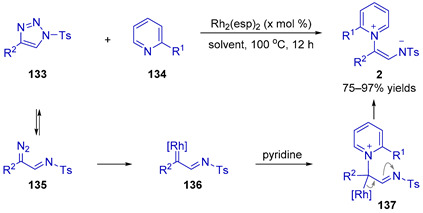							
Rh_2_(esp)_2_ (1 mol %), 1,2-DCE				Rh_2_(esp)_2_ (1.5 mol %), benzene			
Entry	R^1^	R^2^	Yield (%)	Entry	R^1^	R^2^	Yield (%)
1	H	Ph	82	5	Ph	Ph	94
2	Br	Ph	79	6	Ph	4-BrPh	93
3	Me	Ph	88	7	4-FPh	Ph	91
4	Ph	Ph	79	8	3-MePh	Ph	87

**Table 4:** Rh(III)-catalyzed (5 + 2) cyclization of nitrogen-based pyridinium 1,4-zwitterions with alkynes.

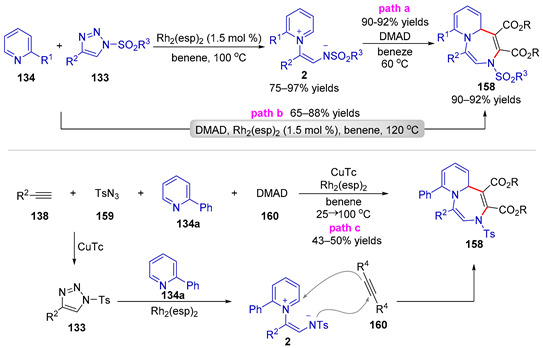							
Entry	R^1^	R^2^	R^3^	R	Yield (%)		
					Path a	Path b	Path c
1	C_6_H_5_	C_6_H_5_	4-MeC_6_H_4_	CH_3_	92	82	50
2	C_6_H_5_	4-MeOC_6_H_4_	4-MeC_6_H_4_	CH_3_	90	79	47
3	C_6_H_5_	4-MeC_6_H_4_	4-MeC_6_H_4_	CH_3_	-	88	43
4	4-ClC_6_H_4_	C_6_H_5_	4-MeC_6_H_4_	CH_3_	-	79	-
5	C_6_H_5_	3-MeOC_6_H_4_	4-MeC_6_H_4_	CH_3_	92	-	-

**Table 5:** Chiral Rh(II)-catalyzed stereoselective (5 + 3) cyclization of nitrogen-based pyridinium 1,4-zwitterion and enol diazoacetate.

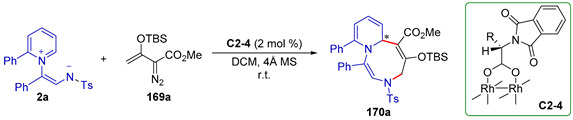			
Entry	[Rh]	Yield (%)	*ee* (%)
1	**C2**/R = *i*Pr	47	62
2	**C3**/R = *t*Bu	23	67
3	**C4**/R = 1-adamantyl	63	90

**Table 6:** Enantioselective cascade cyclization of nitrogen-based quinolinium 1,4-zwitterions with chiral sulfonium ylide salts.

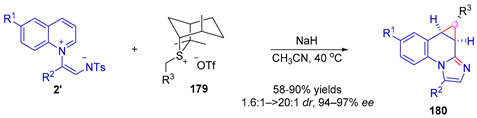						
Entry	R^1^	R^2^	R^3^	Yield (%)	*dr*	*ee* (%)
1	H	C_6_H_5_	C_6_H_5_	65	3.0:1	95
2	Me	C_6_H_5_	C_6_H_5_	66	3.7:1	96
3	H	3-MeC_6_H_4_	C_6_H_5_	65	3.3:1	95
4	H	C_6_H_5_	4-CF_3_C_6_H_4_	88	>20:1	95
5	H	C_6_H_5_	2-MeC_6_H_4_	81	1.6:1	97

**Table 7:** Pd-catalyzed cascade 1,4-dearomative (2 + 3) cycloaddition/intramolecular cyclization of nitrogen-based pyridinium 1,4-zwitterions and TMM.

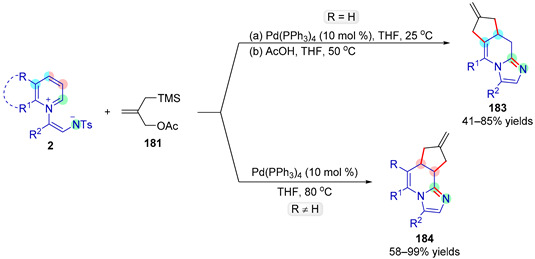					
Entry	R^1^	R^2^	R^3^	Yield (%)	
				183	184
1	H	C_6_H_5_	C_6_H_5_	72	-
2	H	4-MeOC_6_H_4_	C_6_H_5_	56	-
3	H	C_6_H_5_	4-*t*BuC_6_H_4_	52	-
4	Cl	C_6_H_5_	C_6_H_5_	-	99
5	C_6_H_5_	H	C_6_H_5_	-	77

## Data Availability

Not applicable.
